# Genomic surveillance reveals low-level circulation of two subtypes of genogroup C coxsackievirus A10 in Nanchang, Jiangxi Province, China, 2015–2023

**DOI:** 10.3389/fmicb.2024.1459917

**Published:** 2024-09-17

**Authors:** Fenglan He, Chunlong Zhu, Xuan Wu, Liu Yi, Ziqi Lin, Weijie Wen, Chunhui Zhu, Junling Tu, Ke Qian, Qingxiang Li, Guangqiang Ma, Hui Li, Fang Wang, Xianfeng Zhou

**Affiliations:** ^1^Cancer Research Center, Jiangxi University of Chinese Medicine, Nanchang, China; ^2^Jiangxi Provincial Health Commission Key Laboratory of Pathogenic Diagnosis and Genomics of Emerging Infectious Diseases, Nanchang Center for Disease Control and Prevention, Nanchang, China; ^3^The Third Hospital of Nanchang, Nanchang, China; ^4^Jiangxi Provincial Key Laboratory for Diagnosis, Treatment, and Rehabilitation of Cancer in Chinese Medicine, Cancer Research Center, Jiangxi University of Chinese Medicine, Nanchang, China; ^5^Department of Infectious Diseases, Jiangxi Children’s Hospital, Nanchang, China

**Keywords:** coxsackievirus A10, epidemiology, genome, phylogeny, evolutionary dynamics

## Abstract

**Introduction:**

In recent years, coxsackievirus (CV) A10 has been associated with increasing sporadic hand, foot, and mouth disease (HFMD) cases and outbreaks globally. In addition to mild symptoms such as pharyngitis and herpangina, CVA10-related complications or even fatality can occur. Currently, systematic phylogenetic studies of CVA10 are limited.

**Methods:**

In this study, we first explored the epidemiological and genetic characteristics of CVA10 in Nanchang, an inland southeastern city of China, based on the HFMD surveillance network from 2015-2023.

**Results:**

Among 3429 enterovirus-positive cases, 110 (3.04%) were associated with CVA10, with a male-to-female ratio of 1.62. The median age of the CVA10 patients was 2.3 years (interquartile range, IQR 1.0-4.0), with 94.55% (104/110) of the patients aged less than 5 years. Phylogenetic analyses using the full-length VP1, 5’UTR, P1, P2, P3 sequences and near full-length genomes indicated that CVA10 strains (*n* = 57) isolated in Nanchang belonged to genogroup C; two strains identified in 2017 belonged to C1 subtypes clustered with strains from Vietnam, Madagascar, France and Spain; and the others belonged to C2 subtypes interdigitating with CVA10 isolates from mainland China, the United States and Australia. Through extensive analysis, we identified a rare F168Y mutation in epitope 4 of VP1 in a Madagascar strain of genogroup F and a Chinese strain of genogroup C. Based on Bayesian evolutionary analyses, the average nucleotide substitution rate for the VP1 gene of CV10 strains was 3.07×10^–3^ substitutions/site/year. The most recent common ancestor (tMRCA) of genogroup C was dated 1990.84, and the tMRCA of CVA10 strains from Nanchang was dated approximately 2003.16, similar to strains circulating in other regions of China, suggesting that the viruses were likely introduced and cryptically circulated in China before the establishment of the HFMD surveillance network. Recombination analysis indicated intertypic recombination of the Nanchang strain with the genogroup G strain in the 3D region.

**Discussion:**

Given the shifting dominance of viral genotypes and frequent recombination events, the existing surveillance system needs to be regulated to enhance genomic surveillance efforts on a more diverse spectrum of genotypes in the future.

## Graphical abstract

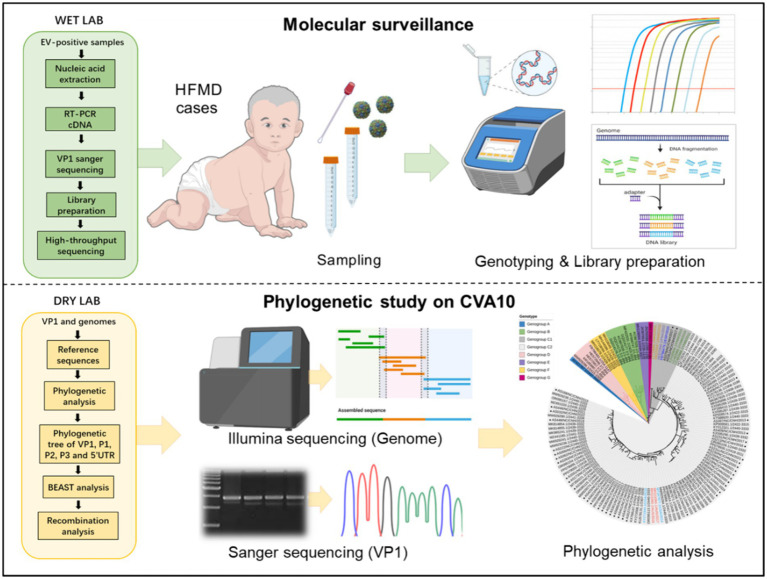

## Introduction

Hand-foot-mouth disease (HFMD) is a common contagious disease caused by enteroviruses that mainly infect children aged younger than 5 years (Aswathyraj et al., 2016). Enterovirus (EV) A71 and coxsackievirus (CV) A16 have been predominant for decades globally and caused the first large-scale HFMD outbreak in the Chinese mainland in 2008 (Xing et al., 2014). Since 2013, HFMD associated with two other serotypes, CVA6 and CVA10, has been frequently reported across China, raising public concern about this emerging pathogen (Yang et al., 2023; Chen et al., 2020; Wang et al., 2023; Sun et al., 2023; Xie et al., 2020). Although CVA6 has become the leading causative agent of HFMD in China, evidence indicates that CVA10 accounts for increasing sporadic HFMD cases and outbreaks worldwide (Yamashita et al., 2005; Mirand et al., 2012; Blomqvist et al., 2010; Sanjay et al., 2022). However, systematic phylogenetic analysis of CVA10 is still limited because the public database contains a limited number of CVA10 genomic sequences. Currently, vaccines or specific anti-CVA10 drugs are unavailable, and fluctuations in the pathogen spectrum pose a challenge for HFMD control and vaccine development.

CVA10 is a member of the EV-A species of the genus *Enterovirus* in the family *Picornaviridae* (Oberste et al., 2004). The viral genome (~7.4 kb) of CVA10 is a positive-sense single-stranded RNA (+ssRNA) comprising a single open reading frame (ORF) flanked by 5′ and 3′ untranslated regions (UTRs). The ORF is divided into three subregions: P1, P2, and P3. P1 encodes four structural proteins, VP1–VP4, whilst seven non-structural proteins 2A–2C and 3A–3D are encoded by P2 and P3, respectively. In general, the VP1 sequence, which encodes major neutralizing epitopes, has been used for genotyping and phylogenetic analysis (Oberste et al., 1999). To date, seven genogroups (A–G) of CVA10 have been assigned based on VP1 variance, and the prototype strain of CVA10 (Kowalik) isolated in America in 1950 formed the independent branch of genogroup A (Ji et al., 2018; Tian et al., 2014). In China, genogroups B and C are the major genogroups of CVA10 according to previous molecular epidemiologic studies (Wang et al., 2022). However, CVA10 was not prioritized for molecular surveillance until the number of HFMD cases associated with non-EV-A71 and non-CVA16 serotypes markedly increased in China in 2013 (He et al., 2021; Guan et al., 2015). To date, there are no data on the genetic features or epidemiological characteristics of CVA10 in Nanchang, the capital of Jiangxi Province in China.

A nationwide molecular surveillance network of EVs was established in 2008 when unprecedented large-scale HFMD outbreaks swept across China (Xing et al., 2014). Our former studies suggested that pathogen spectrum of HFMD in Nanchang substantially changed with C4 EV-A71 predominated during 2010–2012 (Zhou et al., 2015), while D3 CVA6 begin to dominate since 2013 (He et al., 2021). In this study, sporadic HFMD cases (*n* = 5,666) were continuously collected in Nanchang from 2015 to 2023. The epidemiological characteristics and genetic features of CVA10 based on near-full-length genomes (NFLG) were investigated. All representative CVA10 strains circulating in Nanchang belonged to genogroup C, and recombination analysis provided evidence of intertypic recombination with CVA10 genogroup G in the 3D region. Taken together, our findings are the first to clarify the genetic and recombinant features of CVA10 circulating in Nanchang from 2015 to 2023, thereby enriching the global understanding of the CVA10 molecular epidemic.

## Materials and methods

### Sample collection and molecular identification

Clinical samples were obtained from the routine HFMD surveillance network from 2015 to 2023. A total of 5,666 clinical specimens (5,429 pharyngeal/throat swabs, 81 anal swabs and 156 stools) were collected from 5,666 HFMD patients. Swabs were stored in dedicated Universal Transport Medium (UTM) (Yocon, Beijing, China) for transport. Stool samples were diluted to a 10% suspension using MEMs. After thorough mixing, RNA was extracted from 200 μL of each clinical sample using the QIAamp Viral RNA Mini Kit (Qiagen, CA) according to the manufacturer’s instructions. The CVA10, EV-A71, CVA16 and CVA6 viruses were confirmed using commercial real-time RT-PCR kits (No. YJC20107, YJC20101, YJC20102, YJC20103, and YJC20106, respectively) (BioPerfectus, China). Samples positive for universal EVs but negative for the above types were named untyped EVs (UEVs). The use and analysis of all samples in this study were approved by the ethics committee of the Nanchang Center for Disease Control and Prevention, and the procedures were performed according to the approved guidelines.

### Sanger sequencing of full-length VP1

Viral RNA was extracted from CVA10-positive clinical samples using the QIAamp Viral RNA Mini Kit (Qiagen, CA). Commercial real-time PCR kits for pan-EVs were used for a second test to preliminarily evaluate the virus concentration (BioGerm, China). Samples with cycle threshold (Ct) values less than 26 were used for complete genome sequencing, and those with Ct values between 26 and 30 were used for VP1 sequencing. For VP1 gene sequencing, PCR was performed using a PrimeScript One Step RT-PCR Kit (Takara, Japan) and specific primers as previously described (Lu et al., 2012). The PCR products were purified using a QIAquick^®^ PCR Purification Kit (Qiagen, Germany). All the amplicons were sequenced using an ABI 3730 Genetic Analyzer (Thermo Fisher, United States).

### Genome sequencing

For genome sequencing, next-generation sequencing (NGS) was performed. First, cDNA was synthesized using the SuperScript III First-Strand Synthesis System (Invitrogen, United States). Then, nucleic acid enrichment and capture PCR were conducted for each sample using a Tarich Enterovirus Genome Enrichment Kit (BioGerm, China) according to the manufacturer’s instructions. The amplified products were purified using the QIAquick PCR Purification Kit (Qiagen, Germany). The amplification products were mixed at equal concentrations and then sheared into fragments of approximately 200 bp using a Covaris instrument (Covaris Inc., United States). DNA library construction was performed according to the instructions of the Nextera XT DNA Library Preparation Kit (Illumina, United States). Each DNA library was labeled with a different barcode to distinguish each sample used for Illumina sequencing. The DNA libraries were measured with a Qubit, and each library was mixed together at the same concentration. The obtained DNA libraries were qualitatively checked with an Agilent 2100 Bioanalyzer prior to loading onto the Illumina NovaSeq System (Illumina, United States).

### Amino acid mutation sites of CVA10 VP1

Amino acid mutation sites for amino acid sequence logos were generated using the online application WebLogo,^1^ and the VP1 protein structure was predicted using the online tool SWISS-MODEL.^2^ Selection pressure analysis was conducted using Mixed Effects Model of Evolution (MEME) in Datamonkey.^3^

### Phylogenetic analyses

Complete genome sequences of the CVA10 reference strains were obtained from the GenBank database, as listed in Supplementary Table S1, and were aligned pairwise using the ClustalX program. Phylogenetic trees were constructed based on the VP1, 5′ UTR, P1, P2, and P3 regions and the NFGL using the neighbor-joining method with a bootstrap replication of 1,000 cycles in MEGA X software. The online visualization tool iTOL^4^ was used to visualize the phylogenetic tree of VP1.

### Bayesian evolutionary analysis with BEAST

The global evolutionary dynamics of CV10 were inferred based on the entire VP1 region. The Markov chain Monte Carlo (MCMC) method implemented in BEAST (v1.8.4) was used to estimate the divergence time, temporal phylogenies and rates of evolution (Yang et al., 2022). Based on the strict molecular clock model and Bayesian skyline as the preferred population growth model, the posterior probability was analyzed according to the Metropolis-Hastings-Green algorithm as the preferential population growth model, and a Bayesian MCMC run comprised 100 million generations to ensure that each parameter could converge. The output from BEAST was analyzed using TRACER (v1.7.1)^5^ (with estimated sample size (ESS) values higher than 200). When the ESS is greater than 200, the iterative operation is considered to have converged. A maximum clade credibility (MCC) tree was constructed using TreeAnnotator, and the burn-in option was used to remove the first 10% of the sampled trees; the resulting tree was visualized using FigTree (v1.4.4).

To understand the evolution of CA10 strains, we constructed the VP1 gene dataset of CA10 virus from 2008 to 2023, including 57 Nanchang datasets obtained in this study and 348 datasets downloaded from the NCBI database, including 253 cases in mainland China, 2 cases in the Taiwan region, 57 cases in India, 2 cases in the United States, 9 cases in Australia, 10 cases in Vietnam, and 10 cases in the Republic of Madagascar (Supplementary Table S2).

### Recombination analysis

Simplot 3.5.1 software^6^ was used with the default parameter settings to identify potential genetic recombination sites in the viral genome. Bootscan analysis was performed using the neighbor-joining tree model and the Kimura 2-parameter distance algorithm with a window size of 200 nts moving along the alignment in increments of 20 nts with 1,000 resamplings. The PHYLIP internal code (v3.5) (Joseph Felsenstein) and a 70% parental threshold were selected for notification if a region of recombination was detected. The Recombinant Detection Program (RDP) version 4.101 was used to verify natural recombinant strains within complete genome sequence alignments. Seven detection methods including RDP, Geneconv, BootScan, MaxChi, Chimaera, SiScan and 3Seq were conducted. Sequences would be considered as a potential recombinant if at least 3/7 detection methods showed a significant difference (*p*-value <0.05).

## Results

### Epidemiological surveillance of CVA10-related HFMD in Nanchang, 2015–2023

**Figure 1:**
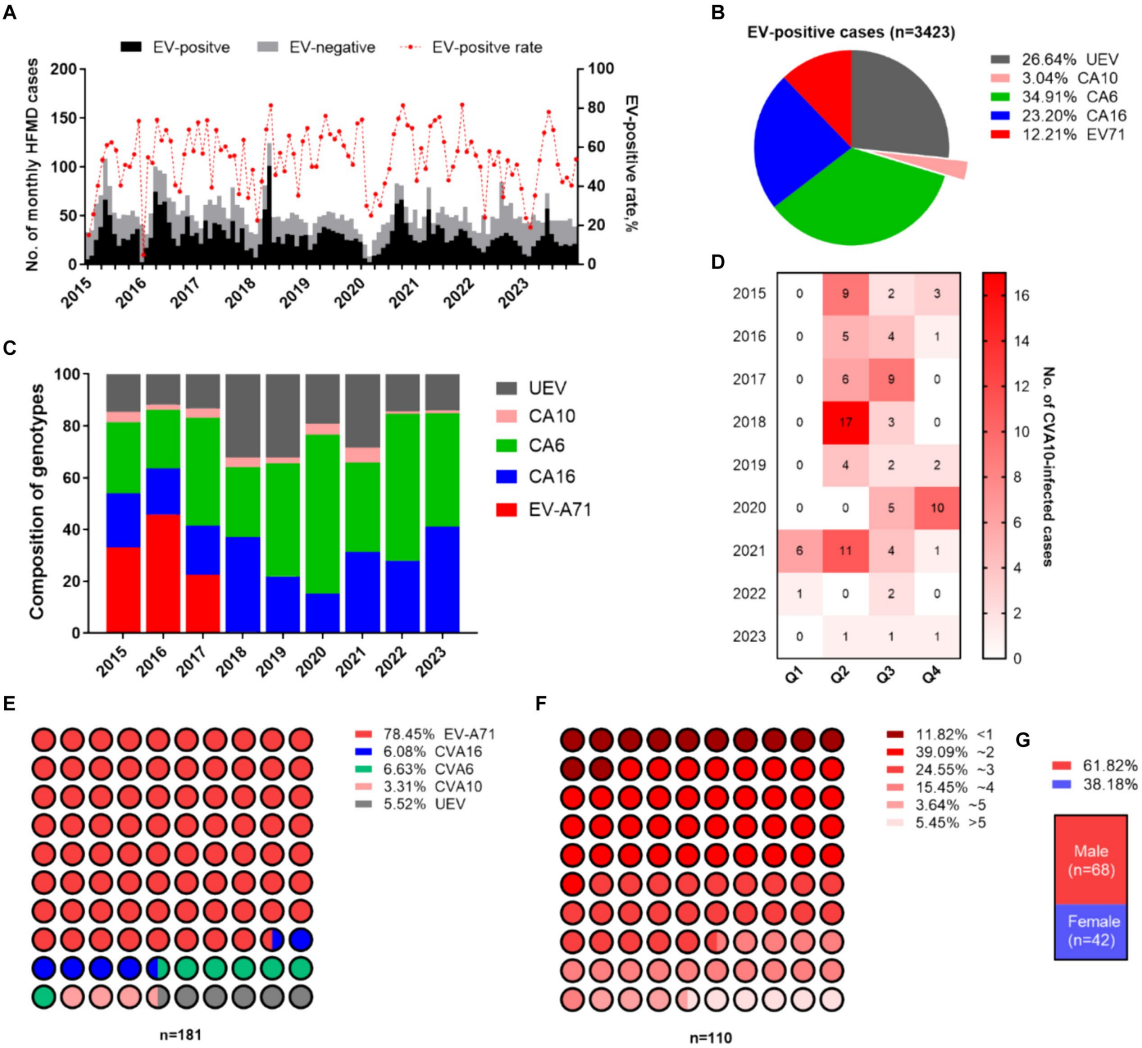
Epidemiological features of HFMD in Nanchang. **(A)** Monthly distribution of HFMD cases during 2015–2023. **(B)** Proportion of serotypes circulating in Nanchang. **(C)** Yearly landscape of the pathogen spectrum of HFMD. **(D)** Seasonality of CVA10 infection. **(E)** Serotype distribution of severe cases. **(F)** Age feature of EV-positive cases. **(G)** Male-to-female ratio of CVA10 cases.

A total of 5,666 sporadic HFMD patients were recruited in Nanchang from January 2015 to December 2023 in this study (Figure 1A). Enteroviruses were identified in 60.50% of the HFMD cases (3,429/5,666). CVA6 has dominated EV-A71 since 2017, accounting for 34.91% (1,195/3,429) of EV-positive cases, followed by CVA16 (23.20%) during 2015–2023 (Figures 1B,C). CVA10 infection accounted for 3.04% of the EV-positive patients (110/3,429). The annual distributions of CVA10-infected individuals were 3.30, 1.73, 3.11, 5.85, 2.30, 4.67, 5.85, 1.08, and 1.08%, respectively, with significant fluctuations from 2015 to 2023 (Figure 1B). Most CVA10-infected cases (85/110, 77.27%) were reported during the second quarter (Q2) and in Q3, which were late spring and early autumn, respectively, in Nanchang (Figure 1D). Moreover, there were no CVA10-positive HFMD cases examined during Q2 of 2020 and 2022, which coincided with nonpharmaceutical interventions (NPIs) against the first two waves of COVID-19 in Nanchang (Zhou et al., 2023). A total of 181 out of 5,666 HFMD patients (3.19%) were diagnosed with severe neurological or cardiopulmonary complications according to the National Health and Family Planning Commission of China. Among the severe cases, 142 were associated with EV-A71 infections (78.45%, 142/181) during 2015–2017 (Figure 1E). Among the 110 CVA10-infected patients, six were diagnosed with severe cases, accounting for 3.31% (6/181) of the reported severe cases (Figure 1E). Most patients with CVA10 infections (94.55%, 104/110) presented mild symptoms. The patients enrolled in this study ranged in age from 3 months to 13 years. The highest incidence rate of CVA10 infection occurred in children aged 1–3 years (63.64%, 70/110) (Figure 1F). The average age of onset was 2.7 years, and the median age was 2.3 years (interquartile range, IQR 1.0–4.0). Specifically, 94.55% (104/110) of the patients were under 5 years old. Children less than 1 year old accounted for 11.82% (13/110) of the patients, and those under 3 years old accounted for 75.55% (83/110) of the patients (Figure 1F). The male-to-female ratio of patients with CVA10 infections reached 1.62 (Figure 1G).

### Phylogenetic analysis

A total of 57 representative strains were used for phylogenetic analyses. Complete VP1 sequencing was conducted for 40 strains, whereas near-complete genome sequencing was conducted for 17 strains. All the sequences were submitted to the GenBank database under the accession numbers PP862409–PP862448 for the VP1 sequences and PP662654–PP662664 and PP862403–PP862408 for the nearly full-length genomes.

**Figure 2:**
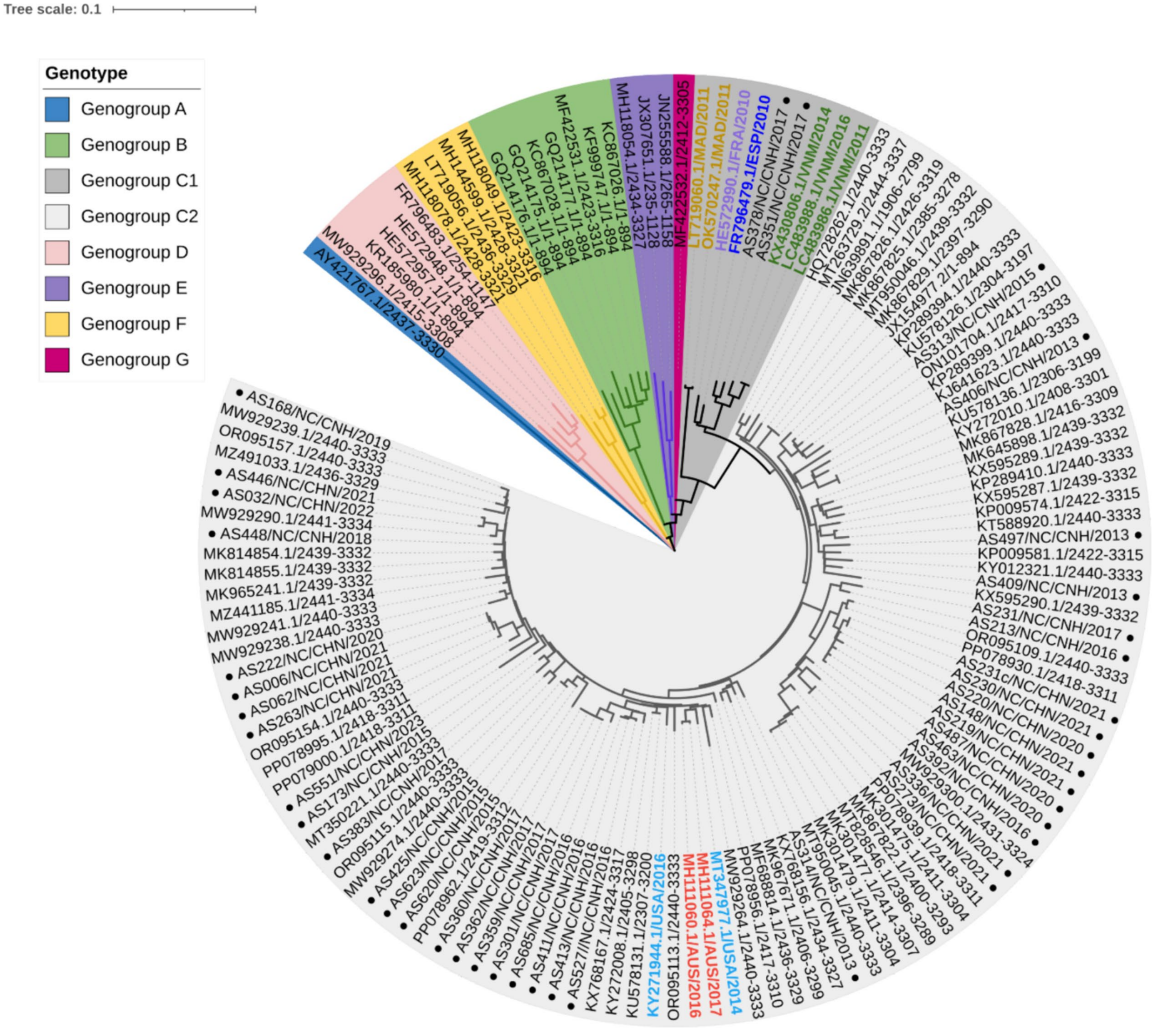
Phylogenetic analysis of CVA10 strains based on complete VP1 sequences (894 bp). Representative reference strains of genogroups A–G (Supplementary Table S1) and Nanchang strains of CVA10 from 2013–2023 (*n* = 40) were included. In genogroup C, strains from outside China were labeled with colored text, with those from Madagascar (MAD) in orange, France (FRA) in purple, Spain (ESP) in dark blue, Vietnam (VNM) in green, the United States of America (USA) in light blue and Australia (AUS) in red. The black text in genogroup C2 shows representative strains from China. ● Strains from Nanchang, Jiangxi Province, China.

To clarify the relationships between Nanchang strains and other representative strains around the globe (Supplementary Table S1), phylogenetic trees were constructed based on nucleotides comprising the VP1 (Figure 2), 5′ UTR, P1 (VP1–VP4), P2 (2A–2C), and P3 (3A–3D) regions and near-complete genome sequences (Supplementary Figures S1, S2). On the basis of the 894 bp VP1 region, CVA10 was assigned to seven genogroups, including genogroup A to genogroup G. The strains in this study belonged to genogroup C and were closely related to CVA10 strains circulating in China, which was supported by phylogenetic analyses of the 5′ UTR, P1, P2, and P3 regions and near-complete genome sequences (Supplementary Figures S1, S2). The genogroup C strains detected in this study were divided into two subtypes, with 2 strains from 2017 clustered into C1 and the others into C2. The VP1 region of these strains shared 91.48–100% nucleotide identity and 96.24–100% amino acid identity. Genogroup C consisted of viruses sampled from various geographic locations worldwide, whereas the CVA10 strains from Nanchang fell within a viral lineage consisting of CVA10 strains from mainland China, Vietnam, Spain, France, the United States, and Australia (Figure 2). As shown in the phylogenetic tree of VP1, the C2 strains in this study were further divided into several subclades interdigitated with CVA10 strains from other provinces of China. All of the CVA10 strains that circulated in different provinces of China correlated well with each other chronologically, suggesting that CVA10 in Nanchang did not evolve independently. Instead, these strains coevolved and cocirculated with those from other provinces, especially neighboring provinces (Guangdong, Zhejiang, Fujian, Anhui) in mainland China. The strains AS378 and AS351 identified in 2017 were closely related to C1 strains isolated in Vietnam during 2011–2016, indicating a possible transmission route for CVA10 from Vietnam to China or common ancestor strains spreading to Vietnam and China.

### Bayesian evolutionary analysis

**Figure 3:**
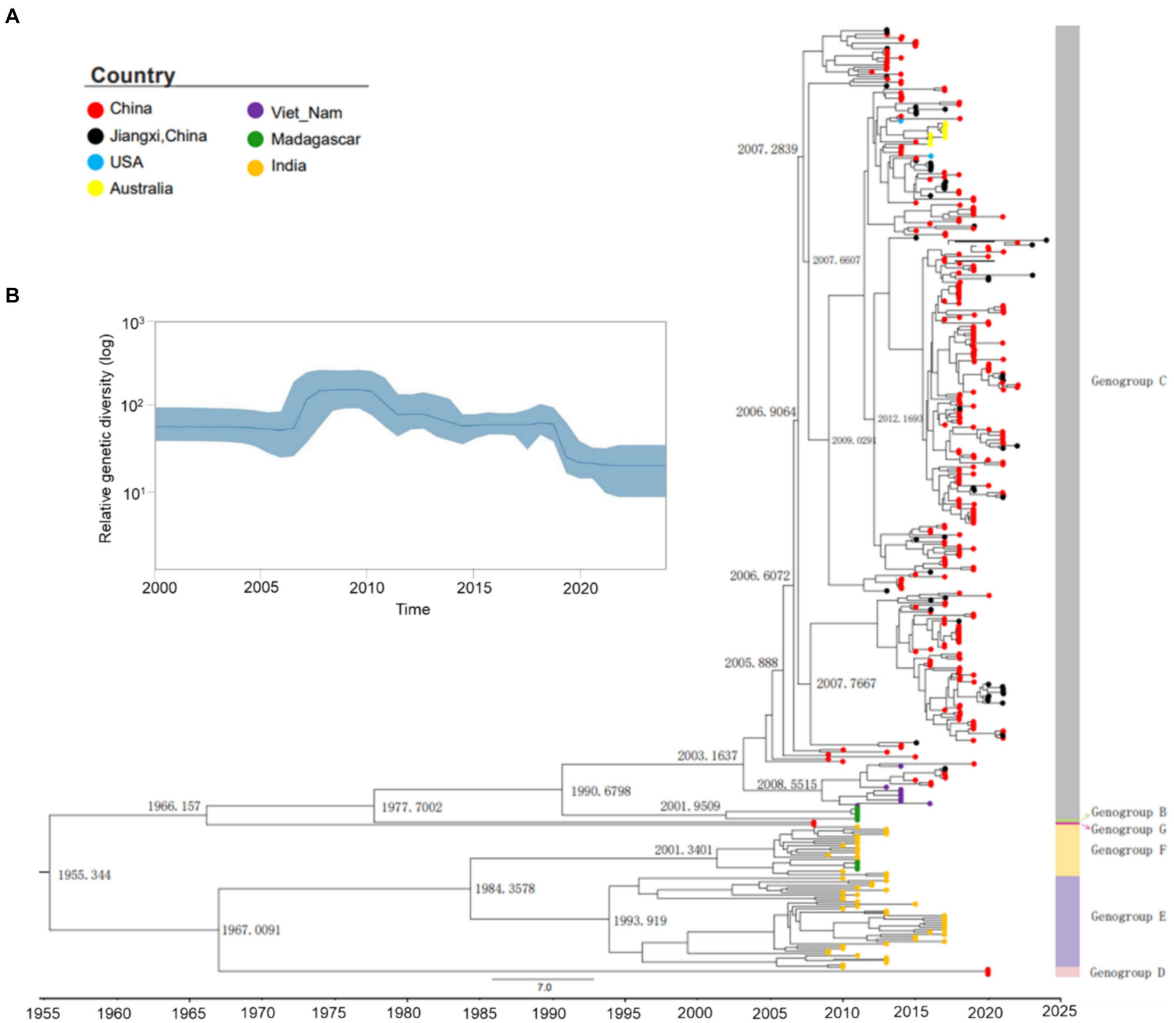
Maximum clade credibility (MCC) tree and Bayesian skyline plot of the analyzed CVA10. **(A)** Maximum clade credibility tree derived from the Bayesian analysis of the VP1 protein of CVA10 with the best fit model (strict molecular clock model), showing the time to the most recent common ancestor (tMRCA) in each principal node. ● Strains of Nanchang, Jiangxi, China. **(B)** Bayesian coalescent inference of genetic diversity and population dynamics using the Bayesian skyline plot available in BEAST 1.8.4. The *x*-axis represents the years of study, and the *y*-axis represents the relative genetic diversity product of the effective population size. The blue line represents the mean estimate, and the light blue shadow represents the 95% HPD.

To investigate the evolutionary history of CVA10, a maximum clade credibility (MCC) tree was constructed based on the entire VP1 sequence of representative CV10 strains (Supplementary Table S2). The average nucleotide substitution rate for the VP1 gene in all CV10 strains worldwide was 3.07 × 10^−3^ substitutions/site/year (95% HPD, 2.80 × 10^−3^–3.34 × 10^−3^). The topological structure of the MCC tree constructed using BEAST was nearly identical to that of the ML tree (Supplementary Figure S4). Based on analysis in the absence of the prototype strain Kowalik (AY421767, United States, 1950), the estimated tMRCA of CVA10 was dated 1955.50, corresponding to the first reported detection of CVA10 in 1950 in the USA. Global CVA10 strains isolated since 1955 formed two branches. Branch 1 included most foreign epidemic strains of genogroups D, E, and F, which arose in 1967. Branch 2 included genogroups B, C, and G, with a tMRCA that emerged in 1966. According to continuous HFMD surveillance, genotype B, which mainly circulated in China, completely disappeared during 2004–2009. The tMRCA of genogroup C dated to 1990.84, suggesting that the virus was likely introduced and cryptically circulated in China before the HFMD cases were well recognized. According to the MCC tree (Figure 3A), genogroup C evolved into C1 and C2 subtypes in approximately 2001–2003, and the earliest strains were reported in Madagascar and Vietnam in 2011 and 2014, respectively.

The Bayesian VP1-based skyline plot of CVA10 viruses sampled across the world revealed fluctuations in the relative genetic diversity of CVA10 from 2007 onward and peaked in approximately 2010 (Figure 3B), highlighting notable changes in viral diversity. This phenomenon coincided with CVA10-associated HFMD epidemics documented in Asia-Pacific regions and Europe, including India in 2009–2010 (Kumar et al., 2013), Thailand in 2008–2013 (Upala et al., 2018), Singapore in 2008 (Wu et al., 2010; Min et al., 2021), and France in 2010 (Mirand et al., 2012).

### Amino acid mutations of VP1 epitopes

**Figure 4:**
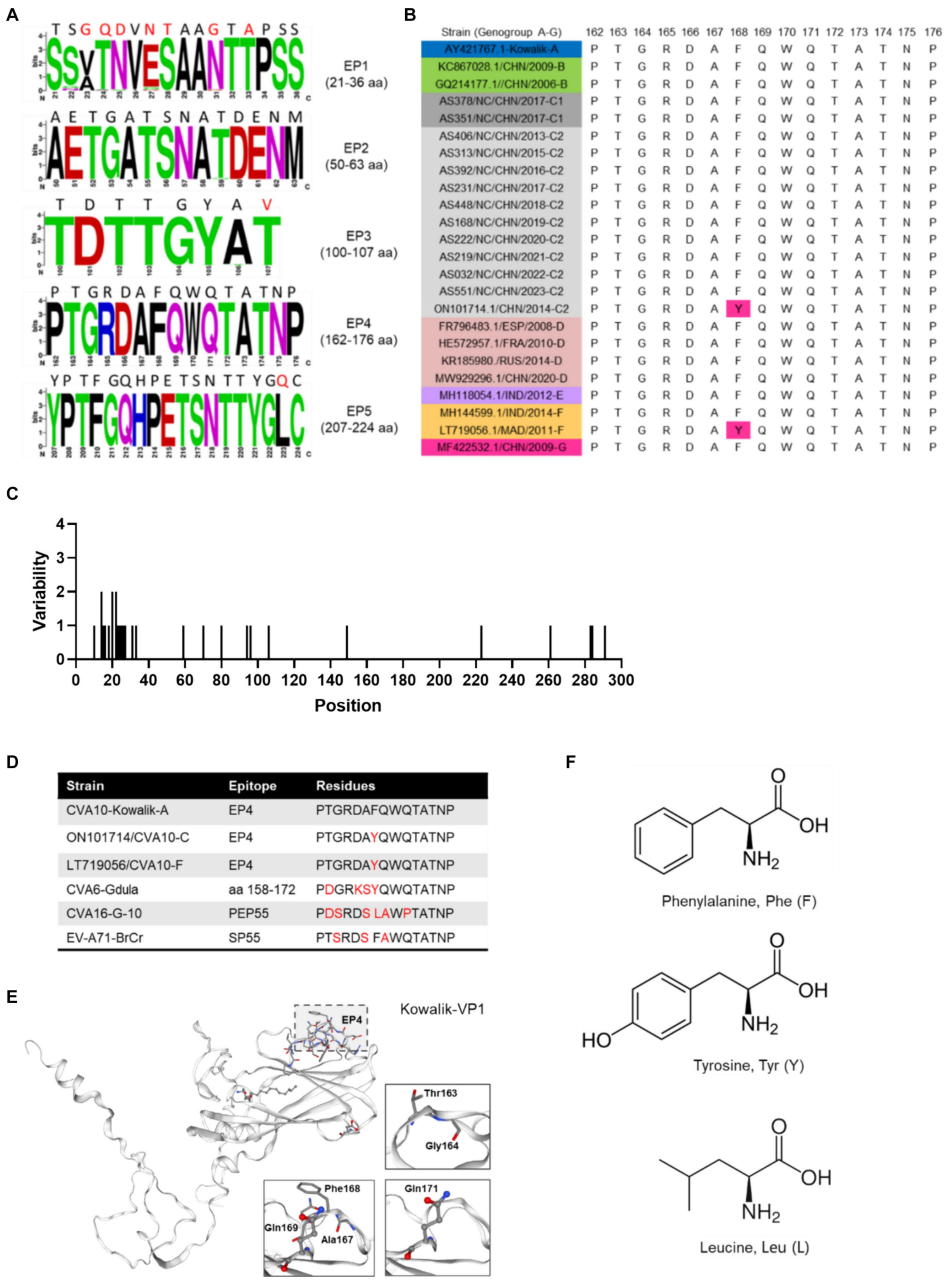
Polymorphism of amino acids of CVA10 VP1 epitopes. **(A)** Amino acid polymorphism of CVA10 VP1 in this study. **(B)** Polymorphism of EP4 amino acids of representative CVA10 strains (genogroups A–G) worldwide. **(C)** Amino acid mutations in VP1 proteins of strains of this study. **(D)** Diversity of aa residues of different serotypes. **(E)** Kowalik VP1 protein structure predicted using SWISS-MODEL with key aa residues (163–164, 167–169, 171) highlighted. **(F)** Three different residue structures in EP4 168 aa of CVA10 VP1 and the corresponding residues in prototype strains CVA6 (Gdula), CVA16 (G-10) and EV-A71 (BrCr). The figures showing amino acid polymorphisms were constructed using weblogo.berkeley.edu/logo.cgi.

Further exploration of the amino acid diversity of the VP1 epitopes (EPs) revealed that most amino acid (aa) mutations in the Nanchang strains occurred in EP1 and differed greatly from those in the prototype strain Kowalik at aa residues 23–25, 27–28, 31 and 33 (Figures 4A,C). A previous study indicated that EP4 (162–176 aa) was a specific linear neutralizing epitope on CV-A10 VP1 and demonstrated potent *in vitro* and *in vivo* neutralization against CVA10 (Zhu et al., 2022). Our data showed that amino acids in EP4 were highly conserved among the representative CVA10 strains of this study in alignment with those of the prototype strain Kowalik (Figures 4A,C). However, by alignment of all worldwide representative strains (Supplementary Table S1), two strains isolated from Madagascar in 2011 and China in 2014 were found to harbor the F168Y mutation (Figure 4B), indicating that monitoring EP4 is necessary for future phylogenetic studies. To explore the corresponding residues in the major serotypes, alignment to prototypes CVA6 (Gdula), CVA16 (G-10) and EV-A71 (BrCr) indicated diversity in numerous aa residues of DE loop (Supplementary Figures S4D–F), suggesting that EP4 is a potential target for monoclonal antibody and vaccine development. Besides, selection pressure analysis using Mixed Effects Model of Evolution (MEME) model indicated that episodic positive or diversifying selection at 284th aa position of VP1, and the fitted tree of global MG94× EV model of codon substitution was showed in Supplementary Figure S5. This result suggests potential selection pressure on the C-terminus of VP1 of CVA10 strains.

### Recombinant features of CVA10 strains in Nanchang

**Figure 5:**
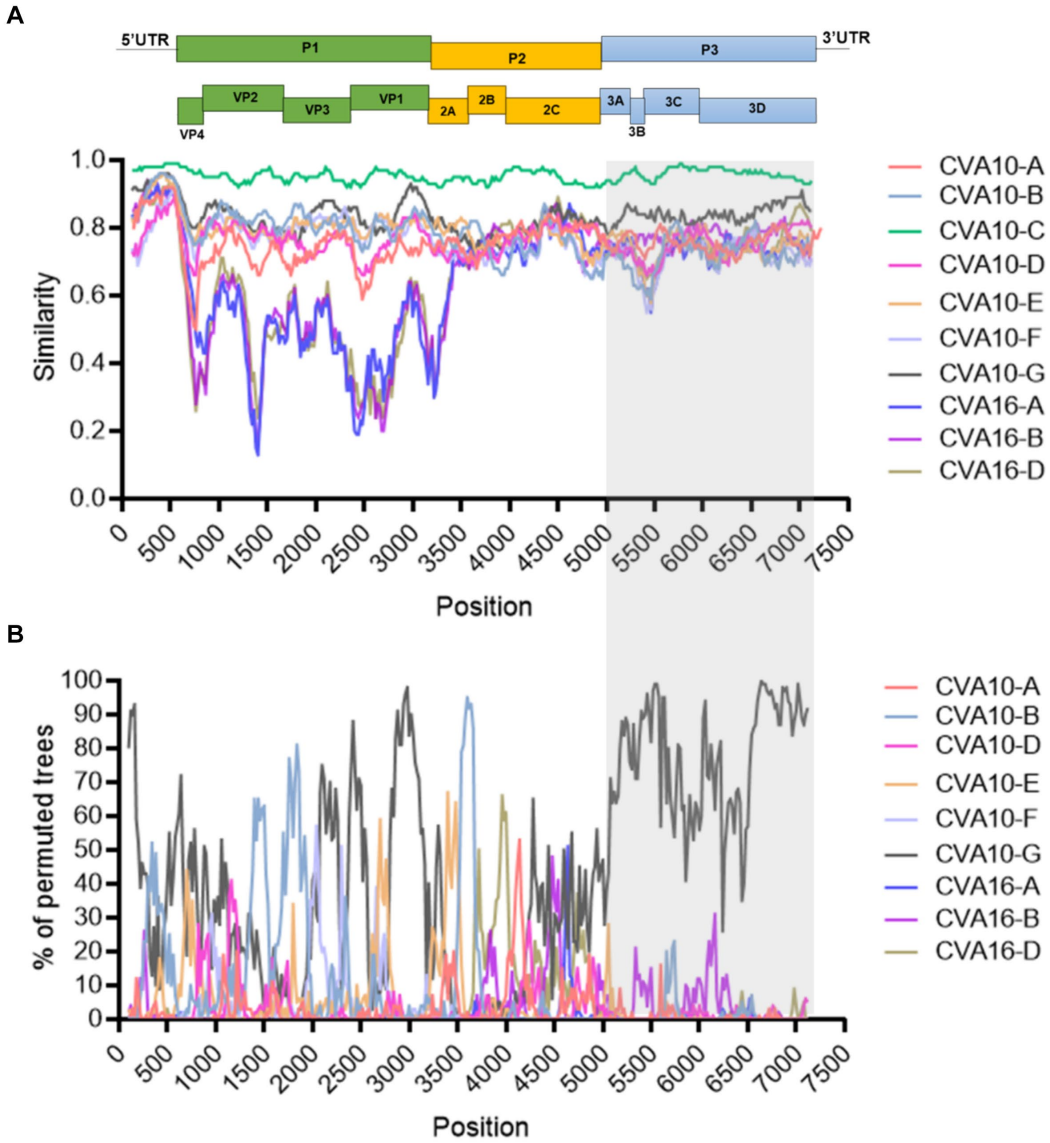
Recombination analysis of representative CVA10 strains identified in Nanchang, Jiangxi Province, China. **(A)** A similarity plot was generated using Simplot (version 3.5.1) with a window size of 200 bp and a step size of 20 bp. **(B)** A bootscan plot was generated using Simplot (version 3.5.1) with a window size of 200 bp and a step size of 20 bp. A total of 1,000 pseudo-replicates using the Kimura 2-parameter model and the neighbor-joining method were used for running the Bootscan analysis. The gray rectangle indicates the recombination region. The representative strains CVA10-A: (AY2714.1), CVA10-B: (MF422531.1), CVA10-D: (MW929296.1), CVA10-E: (MH118054.1), CVA10-F: (MH118049.1), CVA10-G: (MF422532.1), CVA16-A: (U05876.1), CVA16-B: (JX068831.1), CVA16-D: (LT717105.1).

Similarity plot analysis was conducted to investigate the genetic features of the CVA10 genogroup C. Bootscan analysis was also performed to identify potential genetic recombination sites in the viral genomes. The nearly complete genomes of 20 CVA10 isolates (genogroup A, *n* = 1; genogroup B, *n* = 1; genogroup C, *n* = 11; genogroup D, *n* = 1; genogroup F, *n* = 1; genogroup G, *n* = 1) and three CVA16 isolates (genogroup A, *n* = 1; genogroup B, *n* = 1; genogroup D, *n* = 1) were used as reference sequences. AS479/NC/CHN/2017-CVA10-C was chosen as the query when similarity plot analysis and bootscan analysis were conducted (Figure 5). The region located between the 3A gene and the 3D gene (at approximately nucleotide positions of 5,100–7,200 nt) of the CVA10 genogroup C genome was most similar to that of the CVA10 genogroup G (Figure 5A). The bootscan plot showed that the recombination fragments were located between the 3A and 3D regions, suggesting intertypic recombination with CVA10 genogroup G. Phylogenetic analyses of the genomic segments of different regions also supported this finding (Supplementary Figures S1, S3). Strains from Nanchang were clustered with CVA10 isolates in the 5,100–6,500 nt region with two CVA10 genogroup C isolates from Madagascar and one from the genogroup G strain (Supplementary Figure S3A). In the 6,500–7,200 nt region, these strains clustered with CVA10 genogroup G, which is distinct from other genogroups (Supplementary Figure S3B). In the 6,500–7,200 nt region, strains of Nanchang presented the highest homology with the CVA10 genogroup G, which was consistent with the results of the bootscan analysis (Figure 5B). Recombination analysis of CVA10 genogroup C genome with RDP program provided statistical evidence of a recombination breakpoint at 3D gene (Supplementary Table S3). At least four out of seven methods supported this event with *p*-values ranging from 3.19 × 10^−2^ to 6.22 × 10^−5^. The putative major and minor parent strains with best recombination score were CVA10 genogroup C isolate 2015-XMCDC-241 and CVA16 genogroup G isolate MF422532.

## Discussion

As a common infection, HFMD poses a great threat to preschool children and imposes a severe burden on societies, especially in the Asia-Pacific region (Xing et al., 2014; Solomon et al., 2010). In China, EV-A71 and CVA16 were the major EVs responsible for the first large-scale HFMD outbreaks during 2008–2012 (Xing et al., 2014). However, the pathogen spectrum has shifted as the dominance of two major serotypes, EV-A71 and CVA16, has been replaced by that of other EVs, particularly CVA6 (He et al., 2021; Lian et al., 2024; Xiao et al., 2019; Feng et al., 2015). In addition, the promotion of EV-A71 vaccines has accelerated the change in the pathogenic spectrum of EVs (He et al., 2021; Lian et al., 2024). Although CVA6 has been predominant for many years in China, CVA10 has cocirculated at a relatively low level in some cities in China (Zhang et al., 2022). However, continuous genetic surveillance and systematic phylogenetic analysis are still limited in China. In this study, we continuously collected molecular surveillance data of EVs and generated 57 new sequences of CVA10 spanning from 2015–2023, which provided valuable information on the evolution of the virus. Our study revealed that low-level circulation of CVA10 (~3% of EV-positive cases) occurred mainly among <3-year-old children from May–September in Nanchang (Figure 1D). The epidemiological features of CVA10 identified in this study were similar to those reported in previous publications from Shanghai (Wang et al., 2022), Zhejiang (Sun et al., 2023) and Shanxi (Wang et al., 2023). However, CVA10 was reported to cause as many as 25% of HFMD cases in Guangzhou in 2018 (Xie et al., 2020) and 41% of cases in Wuhan from 2012–2013 (Yang et al., 2015). These observations indicated a diverse pathogen spectrum in different regions of China.

Generally, CVA10 infections manifest as benign illnesses such as pharyngitis and herpangina, while CVA10-related complications or even fatality can occur (Tian et al., 2014). As the serotype spectrum of non-EV-A71/CVA16-associated infections was unclear during the first large-scale HFMD outbreaks across China during 2008–2012 (Xing et al., 2014), it was difficult to discern the contribution of CVA10 to severe and fatal cases during this period. Our study revealed that 3.31% (4/110) of severe cases were caused by CVA10, which was slightly greater than that reported in 0.36% (1/277) of Shanghai during 2016–2020 (Wang et al., 2022) and 0.25% in Jinan (1/400) during 2009–2013 (Guan et al., 2015) but significantly lower than that previously reported in 39% (7/18) in Xiamen in 2015 (Chen et al., 2017). However, the limited number of cases could lead to biased results. Therefore, it is particularly important to strengthen the genomic epidemiology and symptom surveillance of CVA10.

In this study, genotyping based on the entire VP1 gene and phylogenetic analysis of the NFGL were performed. The strains identified in Nanchang belonged to genogroup C, in which 2 strains was concentrated in the C1 subtype and the other 55 in the C2 subtype. The pairwise distances of the 5′ UTR, P1 region, P2 region, P3 region and NFLG of the Nanchang strains were 94.5–99.2%, 91.1–97.6%, 91.4–97.6%, 91.7–99.6% and 90.9–97.5%, respectively. Currently, genogroup C is the predominant subtype of CVA10 circulating in China, with sporadic cases associated with genogroup F in Zhejiang (Sun et al., 2023) and Guangdong (Lian et al., 2024) and genogroup D in Shanghai (Wang et al., 2022). Notably, for the first time, genogroup D, which has mainly been reported in Europe, emerged in mainland China (Wang et al., 2022). These findings suggest multiple transmission routes of CVA10 in China or cross-border transmission of different genogroups. Phylogenetic studies indicated that genogroup C2 was the predominant subtype circulating around mainland China (Ji et al., 2018; Lian et al., 2024), while very few cases were associated with C1 subtypes in the current study and in previous publications (Wang et al., 2023). According to the MCC tree (Figure 3A), genogroup C evolved into C1 and C2 subtypes between 2001 and 2003, and the earliest strains were reported in Madagascar and Vietnam in 2011 and 2014, respectively. This result indicated that C1 subtypes might have undergone cryptically circulation and cross-border transmission since the 2000s. Recombination is known to be frequent among species of EVs, which promotes the spatiotemporal evolution and differentiation of EVs and subsequently leads to the localization and epidemicity of the viruses (Wang et al., 2022; Lian et al., 2024; Tomba Ngangas et al., 2022). Generally, recombination events occur mainly in nonstructural protein-coding regions of EVs (Puenpa et al., 2022). Researchers have demonstrated that the 3D^pol^ error-prone RNA-dependent RNA polymerases (RdRps) of EVs lack proofreading function, leading to misincorporations of 10^−5^–10^−3^ per nucleotide site during genome replication (Domingo and Holland, 1997), while recombination can prevent deleterious mutation accumulation. Our study revealed that CVA10 strains circulating in Nanchang exhibited segment recombination the 3D region, in which mutation or recombination may be associated with different clinical manifestations (Ogi et al., 2017). Therefore, it is necessary for continuous genomic surveillance of CVA10, with a focus on phylodynamic, recombination events and viral virulence in the future.

The VP1 protein, the major immunogen of EVs, contains multiple epitopes. In this study, a comparison of the amino acid sequences of the VP1 proteins revealed 96.24–100% homology. Five epitopes were identified in the CVA10 VP1 protein, and epitope 4 (162–176 residues) located in the EF loop is a specific linear neutralizing epitope that effectively neutralizes CVA10 (Zhu et al., 2022). Amino acid sequence analysis of CVA10 genotypes A–G, including Nanchang strains, revealed that this linear neutralizing epitope was highly conserved in CVA10, except for a rare mutation, F168Y, which was harbored in only two strains of genogroups C and F, which led us to speculate whether this variation is related to receptor binding, selection pressure or immune evasion. Recently, KREMEN1 was identified as a host entry receptor that interacts with VP1, VP2, and VP3 of CVA10 (Staring et al., 2018; Cui et al., 2020) and might also be responsible for virion attachment and uncoating (Cui et al., 2020). To fully understand these functional differences, an integrated analysis is needed to explore the impact of F168Y on virus–host interactions.

This study has some limitations. The epidemiological features of CV10 before 2015 was unclear due to the limited coverage of two major serotypes EV-A71 and CVA16. Despite the optimization of the surveillance system and the inclusion of CVA6 and CVA10 since 2015, there is still ~20% EV-positive cases to be identified. Given the shifting dominance of viral genotypes and frequent recombination events, it is needed to regulate existing surveillance system to enhance genomic surveillance efforts on a more diverse spectrum of genotypes in the future.

## Data Availability

The datasets presented in this study can be found in online repositories. The names of the repository/repositories and accession number(s) can be found below: https://www.ncbi.nlm.nih.gov/genbank/, PP862409–PP862448; https://www.ncbi.nlm.nih.gov/genbank/, PP662654–PP662664; https://www.ncbi.nlm.nih.gov/genbank/, PP862403–PP862408.

## Footnotes

^1^ https://www.weblogo.threeplusone.com

^2^ https://swissmodel.expasy.org/

^3^ https://www.datamonkey.org/

^4^ https://itol.embl.de

^5^ http://beast.community/tracer

^6^ http://sray.med.som.jhmi.edu/SCRoftware/simplot/
