# Supplementary material for: Genomic surveillance reveals low-level circulation of two subtypes of genogroup C coxsackievirus A10 in Nanchang, Jiangxi Province, China, 2015–2023
Source: Front Microbiol. 2024 Sep 17;15:1459917. doi: 10.3389/fmicb.2024.1459917 (PMC11443423; doi:10.3389/fmicb.2024.1459917)
Supplement: Supplementary file 1 [file Data_Sheet_1.pdf]

Supplementary Data

Genomic surveillance reveals low-level circulation of two subtypes of genogroup C coxsackievirus A10 in Nanchang, Jiangxi Province, China, 2015-2023

Fenglan He[1,2#], Chunlong Zhu[3#], Xuan Wu[1#], Liu Yi[2], Ziqi Lin[1], Weijie Wen[1,4], Chunhui Zhu[5], Junling Tu[2], Ke Qian[2], Qingxiang Li[3], Guangqiang Ma[1], Hui Li[2], Fang Wang[1,4*], Xianfeng Zhou[1,2,4*]

1 Cancer Research Center, Jiangxi University of Chinese Medicine, Nanchang, China
2 Jiangxi Provincial Health Commission Key Laboratory of Pathogenic Diagnosis and Genomics of Emerging Infectious Diseases, Nanchang Center for Disease Control and Prevention, Nanchang, China
3 The Third Hospital of Nanchang, Nanchang, China
4 Jiangxi Provincial Key Laboratory for Diagnosis, Treatment, and Rehabilitation of Cancer in Chinese Medicine, Cancer Research Center, Jiangxi University of Chinese Medicine, Nanchang, China
5 Department of Infectious Diseases, Jiangxi Children’s Hospital, Nanchang, China

[#] These authors contributed equally to this work
* Correspondence and requests for materials should be addressed to XZ (email: nccdczxf@126.com) or FW (20040807@jxutcm.edu.cn)

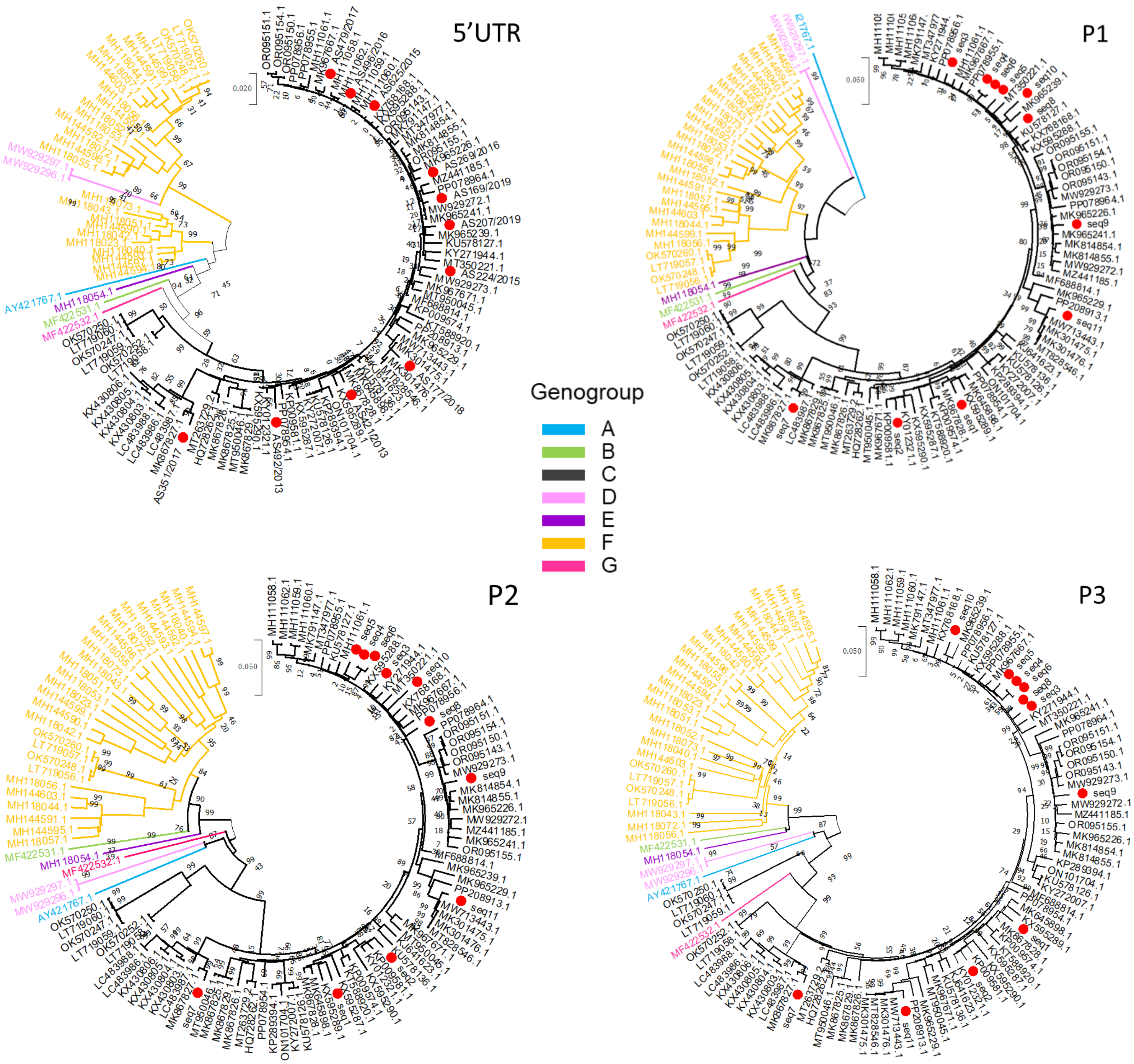

Supplementary Fig. S1 Phylogenetic analysis of CVA10 isolates based on 5′UTR, P1, P2 and P3 regions. The phylogenetic dendrogram was constructed by the neighbor-joining method and validated with 1000 replicates. Sequences available from GenBank database were listed in Supplementary Table S1. Sequences of CVA10 genogroup A is indicated in blue, genogroup B in green, genogroup C in black, genogroup D in pink, genogroup E in purple, genogroup F in yellow and genogroup G in red. Sequences in this study are labeled with solid red circle.

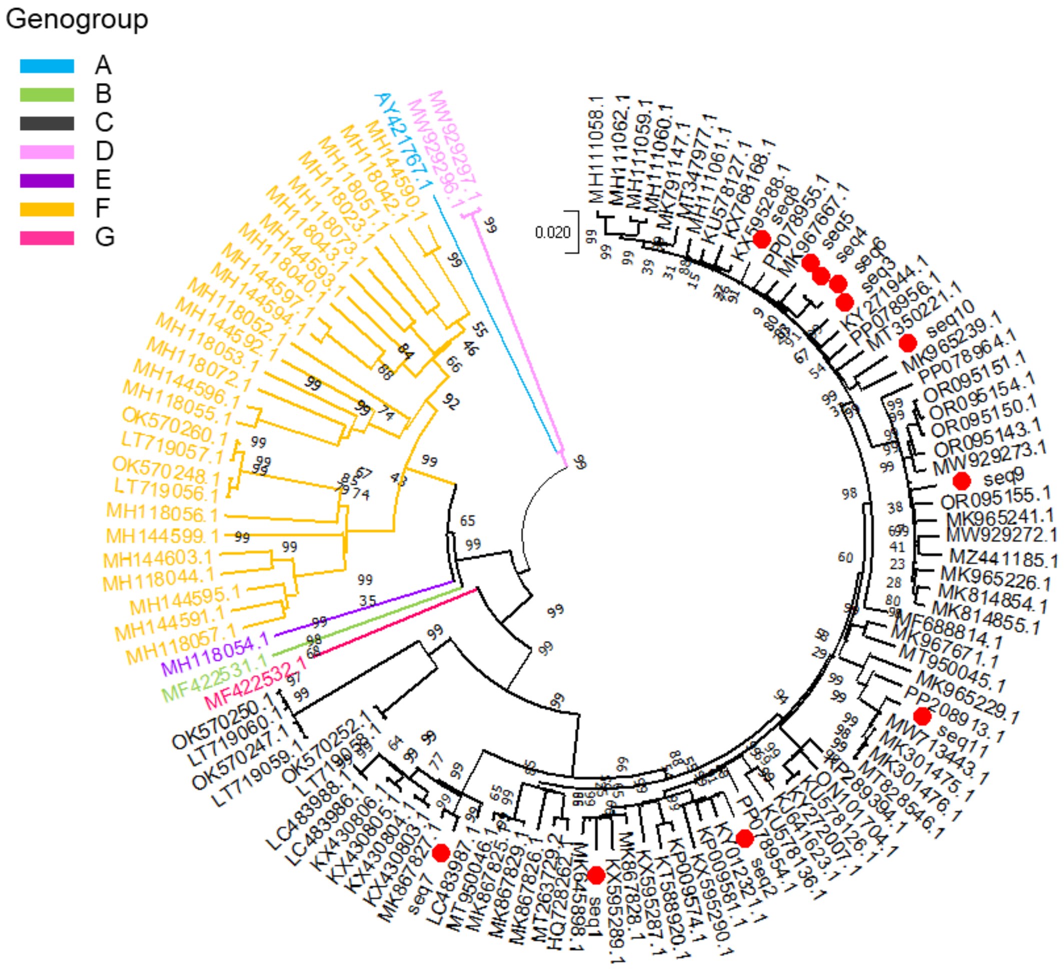

Supplementary Fig. S2 Phylogenetic analysis of CVA10 isolates based on near full-length genome. The phylogenetic dendrogram was constructed by the neighbor-joining method and validated with 1000 replicates. Sequences available from GenBank database were listed in Supplementary Table S1. Sequences of CVA10 genogroup A is indicated in blue, genogroup B in green, genogroup C in black, genogroup D in pink, genogroup E in purple, genogroup F in yellow and genogroup G in red. Sequences in this study are labeled with solid red circle.

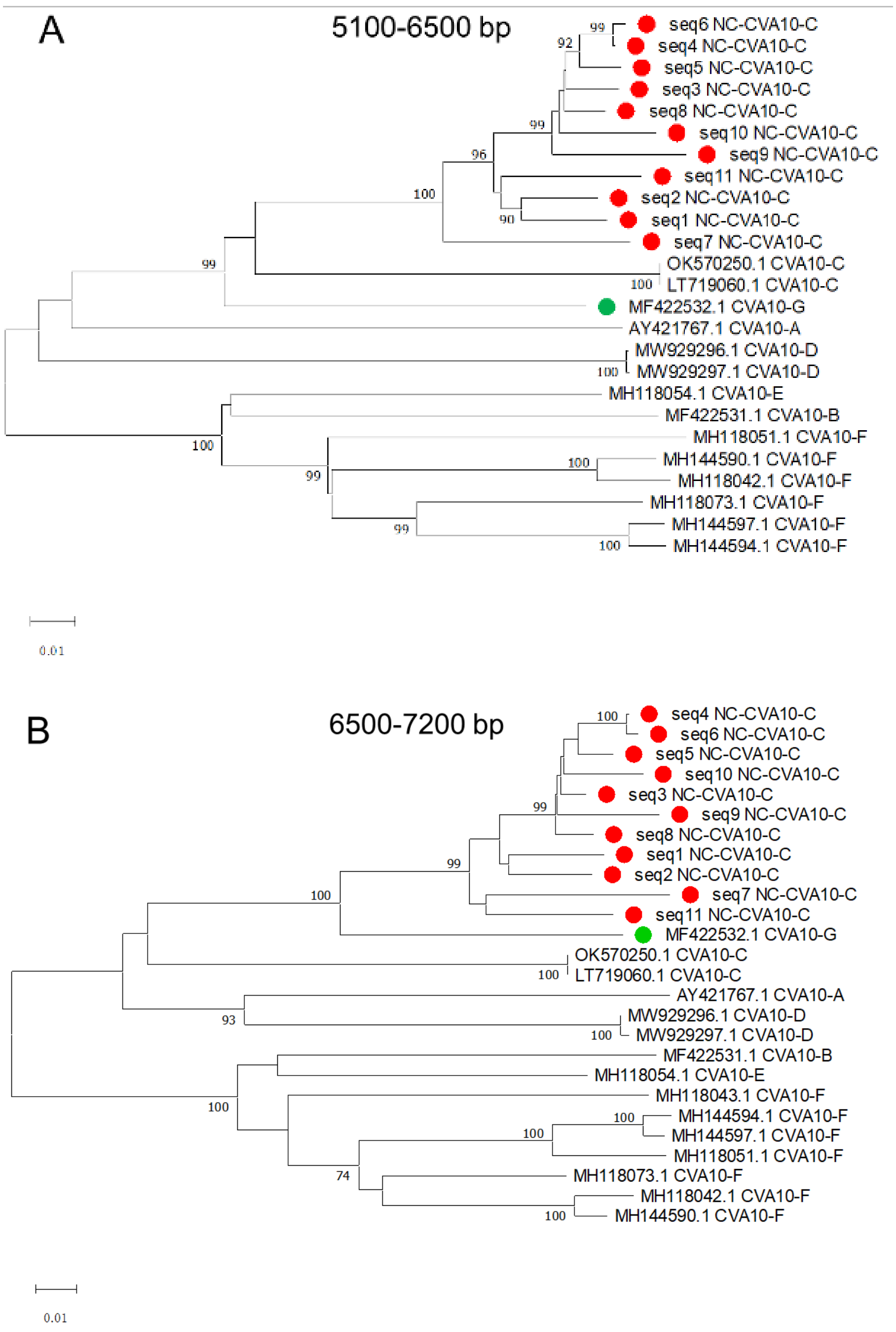

Supplementary Fig. S3 Phylogenetic analyses of the potential recombinant regions. **A** 5100–6500bp region. **B** 6500–7200bp region. The phylogenetic dendrogram was constructed by the neighbor-joining method and validated with 1000 replicates. Only bootstrap values over 70% are shown. The reference sequences were consisted of a dataset of 26 CVA10 genomes (genogroup A, n=1; genogroup B, n=1; genogroup C, n=13; genogroup D, n=2; genogroup E, n=1; genogroup F, n=7; genogroup G, n=1). Strains of Nanchang were labeled with solid red circles. Genogroup G (MF422532) was labeled with solid green circle, CV, coxsackievirus.

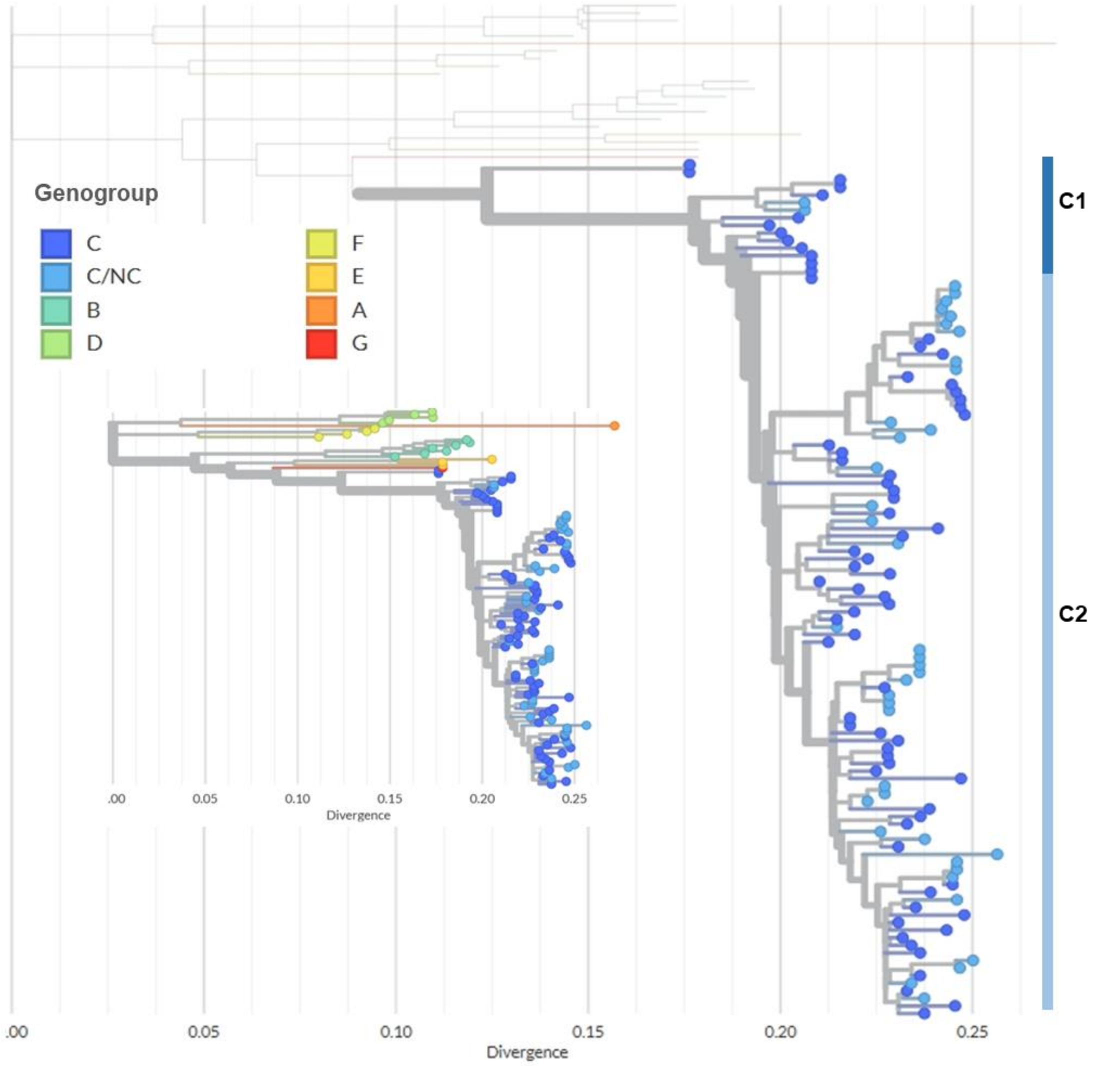

Supplementary Fig. S4 Maximum likelihood tree of CVA10 constructed based on VP1 sequences. The zoomed-out image illustrated the time scaled phylogenetic tree of VP1 of genogroup C. C/NC: Strains of Nanchang. The annotated data and metadata were compiled and exported in JSON format for visualization in the Auspice interactive phylodynamic tool (https://auspice.us/).

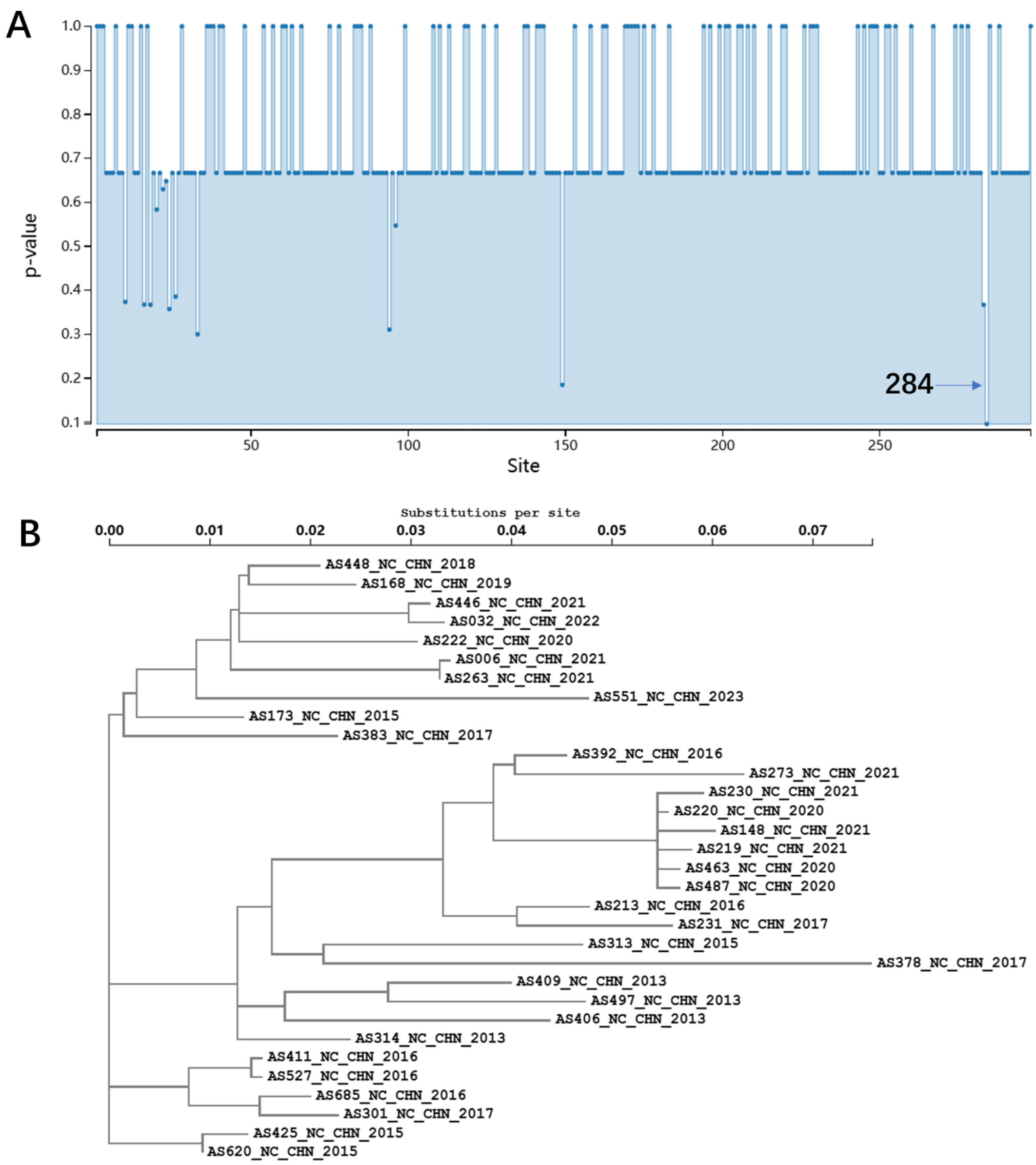

Supplementary Fig. S5 Selection pressure analysis was conducted using Mixed Effects Model of Evolution (MEME) in Datamonkey. **A** evidence indicated that episodic positive/diversifying selection at 284 aa position with p-value threshold of 0.1. **B** the fitted tree on the basis of the global MG94× EV model of codon substitution.

Supplementary Table S1 Reference sequences for CVA10 genotyping and phylogenetic analysis

Serial No.	Accession No.	Strain name	Genogroup	Year	Country
1	AY421767.1	Kowalik	A	1950	USA
2	KC867026.1	JB143090155	B	2009	China
3	KF999747.1	HuN09-22	B	2009	China
4	GQ214177.1	06251/SD/CHN/2006/CA10	B	2006	China
5	GQ214176.1	04294/SD/CHN/2004/CA10	B	2004	China
6	GQ214175.1	H587F/SD/CHN/2008/CA10	B	2008	China
7	MF422531.1	61216-2814	B	2008	China
8	MH118054.1	12-2067-2	B	2012	India
9	PP078930.1	611_GD/CHN_2018	C	2018	China
10	PP078939.1	490_GD/CHN_2016	C	2016	China
11	PP078956.1	1660_GD/CHN_2015	C	2015	China
12	PP078962.1	485_GD/CHN_2018	C	2018	China
13	PP078995.1	92_GD/CHN_2021	C	2021	China
14	PP079000.1	434_GD/CHN_2020	C	2020	China
15	OR095109.1	ZJ-19-168	C	2019	China
16	OR095113.1	ZJ-17-189	C	2017	China
17	OR095115.1	ZJ-18-7	C	2018	China
18	OR095154.1	ZJ-22-55	C	2022	China
19	OR095157.1	ZJ-19-169	C	2019	China
20	MZ441185.1	3R3/YN/CHN/2019	C	2019	China
21	MT950045.1	A72/YN/CHN/2015	C	2015	China
22	MT950046.1	R09191/YN/CHN/2009	C	2009	China
23	MT828546.1	V6-19/XY/CHN/2017-P25	C	2015	China
24	MW929238.1	BS17-78	C	2017	China
25	MW929239.1	CN18-124	C	2018	China
26	MW929241.1	FX17-257	C	2017	China
27	MW929264.1	JD19-89	C	2019	China
28	MW929274.1	PD17-9	C	2017	China
29	MW929290.1	SJ18-60	C	2018	China
30	MW929300.1	YP19-86	C	2019	China
31	MZ491033.1	CVA10/Wuxi83/China/2019	C	2019	China
32	ON101704.1	YNKG1-7	C	2014	China
33	MK867822.1	V4-9/XY/CHN/2017	C	2017	China
34	MK867825.1	R09191/YN/CHN/2009	C	2009	China
35	MK867826.1	K975/YN/CHN/2010	C	2010	China
36	MK867828.1	K136/YN/CHN/2013	C	2013	China
37	MK867829.1	R103/YN/CHN/2015	C	2015	China
38	MK967671.1	TA137R	C	2014	China
39	MT350221.1	CA10/JX2545/2017	C	2017	China
40	MT263729.2	CV-A10-HB09-035	C	2009	China
41	MK965241.1	HEV793797	C	2018	China
42	LC483986.1	HVN11_028_Bac_GiangVNM/2011	C	2011	Viet Nam

43	LC483988.1	HVN16_073_Hai_PhongVNM/2016	C	2016	Viet Nam
44	KX430806.1	CVA10/Homo sapiens/VNM/6.2/2014	C	2014	Viet Nam
45	KX430810.1	CVA10/Homo sapiens/VNM/8.2/2014	C	2014	Viet Nam
46	MK301475.1	R6-19/XY/CHN/2017	C	2017	China
47	MK301477.1	V6-19/XY/CHN/2017-P5	C	2018	China
48	MK301479.1	V6-19/XY/CHN/2017-P15	C	2018	China
49	MK814854.1	HEV9667699	C	2018	China
50	MK814855.1	HEV792611	C	2018	China
51	MT347977.1	USA/2014-23299	C	2014	USA
52	MK645898.1	P148/ZS/CHN/2012	C	2012	China
53	LT719060.1	MAD-9937-11	C	2011	Madagascar
54	OK570247.1	MAD-9856-2011	C	2011	Madagascar
55	MH111060.1	C136/CHW/AUS/2016	C	2016	Australia
56	MH111064.1	C150/CHW/AUS/2017	C	2017	Australia
57	KY271944.1	USA/TN/2016-OB2038	C	2016	USA
58	KX595287.1	CVA10/Shenzhen152/CHN/2013	C	2013	China
59	KX595289.1	CVA10/Shenzhen180/CHN/2014	C	2014	China
60	KX595290.1	CVA10/Shenzhen10/CHN/2015	C	2015	China
61	KY012321.1	CVA10-FJ-01	C	2014	China
62	KX768156.1	2014-XMCDC-361-CA10	C	2014	China
63	KX768167.1	2015-XMCDC-241-CA10	C	2015	China
64	KY272008.1	QD102R/Shandong/China/2014	C	2014	China
65	KY272010.1	HZ302R/Shandong/China/2014	C	2014	China
66	KT588920.1	CV-CNIC/Guizhou/CHN/14	C	2014	China
67	KU578126.1	Heze/SD/CHN/2014	C	2014	China
68	KU578131.1	Taian2/SD/CHN/2014	C	2014	China
69	KU578136.1	Qingdao2/SD/CHN/2014	C	2014	China
70	KP289394.1	CV-A10/P1005/2013/China	C	2013	China
71	KP289399.1	CV-A10/P638/2013/China	C	2013	China
72	KP289410.1	CV-A10/P978/2013/China	C	2013	China
73	KP009574.1	FY01/AH/CHN/2013	C	2013	China
74	KP009581.1	FY08/AH/CHN/2013	C	2013	China
75	KJ641623.1	CVA10/JB141310010	C	2013	China
76	HQ728262.1	CVA10/SD/CHN/09	C	2019	China
77	MW929296.1	SJ20-7	D	2020	China
78	FR796483.1	ESP08/54602	D	2008	Spain
79	HE572948.1	CF142021_FRA10	D	2010	France
80	HE572957.1	CF159016_FRA10	D	2010	France
81	KR185980.1	SPb_4982/13EVI/Nov-29/14/RU	D	2013	Russia
82	MH118054.1	12-2067-2	E	2012	India
83	JN255588.1	CAF-OMB-06-062	E	2006	Central African Republic
84	JX307651.1	T08-235	E	2008	Chad
85	LT719056.1	MAD-3995-11	F	2011	Madagascar
86	MH118049.1	13-1512-1	F	2013	India
87	MH118078.1	10-3408-2	F	2010	India
88	MH144599.1	13-2380-2_A10	F	2013	India

89	MH118039.1	17-118-2	F	2017	India
90	MH118040.1	16-2922-1	F	2016	India
91	MH118042.1	15-3903-1	F	2015	India
92	MH118089.1	09-1701-1	F	2009	India
93	MH144590.1	15-2711-1	F	2015	India
94	MH144591.1	11-4519-1	F	2011	India
95	MH144592.1	13-2591-2	F	2013	India
96	MH144593.1	17-2007-2	F	2017	India
97	MF422532.1	61217-2276	G	2008	China

Supplementary Table S2 Metadata of CVA10 strains selected for BEAST analysis

Serial No.	Strain name/accession No.	Date of collection	Location
1	AS228/NC/CHN/2021_China:Jiangxi_2021-05-15	2021/5/15	China:Jiangxi
2	AS516/NC/CHN/2020_China:Jiangxi_2020-12-19	2020/12/19	China:Jiangxi
3	AS223/NC/CHN/2020_China:Jiangxi_2020-08-14	2020/8/14	China:Jiangxi
4	AS090/NC/CHN/2024_China:Jiangxi_2024-03-11	2024/3/11	China:Jiangxi
5	AS366/NC/CHN/2023_China:Jiangxi_2023-08-28	2023/8/28	China:Jiangxi
6	AS331/NC/CHN/2021_China:Jiangxi_2021-07-13	2021/7/13	China:Jiangxi
7	AS220/NC/CHN/2020_China:Jiangxi_2020-08-01	2020/8/1	China:Jiangxi
8	AS222/NC/CHN/2020_China:Jiangxi_2020-08-12	2020/8/12	China:Jiangxi
9	AS463/NC/CHN/2020_China:Jiangxi_2020-12-01	2020/12/1	China:Jiangxi
10	AS487/NC/CHN/2020_China:Jiangxi_2020-12-14	2020/12/14	China:Jiangxi
11	AS006/NC/CHN/2021_China:Jiangxi_2021-01-19	2021/1/19	China:Jiangxi
12	AS062/NC/CHN/2021_China:Jiangxi_2021-02-03	2021/2/3	China:Jiangxi
13	AS148/NC/CHN/2021_China:Jiangxi_2021-04-06	2021/4/6	China:Jiangxi
14	AS219/NC/CHN/2021_China:Jiangxi_2021-04-24	2021/4/24	China:Jiangxi
15	AS230/NC/CHN/2021_China:Jiangxi_2021-05-05	2021/5/5	China:Jiangxi
16	AS231c/NC/CHN/2021_China:Jiangxi_2021-05-17	2021/5/17	China:Jiangxi
17	AS263/NC/CHN/2021_China:Jiangxi_2021-05-06	2021/5/6	China:Jiangxi
18	AS273/NC/CHN/2021_China:Jiangxi_2021-06-07	2021/6/7	China:Jiangxi
19	AS336/NC/CHN/2021_China:Jiangxi_2021-07-10	2021/7/10	China:Jiangxi
20	AS446/NC/CHN/2021_China:Jiangxi_2021-09-16	2021/9/16	China:Jiangxi
21	AS032/NC/CHN/2022_China:Jiangxi_2022-01-06	2022/1/6	China:Jiangxi
22	AS551/NC/CHN/2023_China:Jiangxi_2023-12-19	2023/12/19	China:Jiangxi
23	AS168/NC/CHN/2019_China:Jiangxi_2019-05-01	2019/5/1	China:Jiangxi
24	AS169/NC/CHN/2019_China:Jiangxi_2019-11-21	2019/11/21	China:Jiangxi
25	AS173/NC/CHN/2015_China:Jiangxi_2015-04-20	2015/4/20	China:Jiangxi
26	AS177/NC/CHN/2018_China:Jiangxi_2018-01-02	2018/1/2	China:Jiangxi
27	AS207/NC/CHN/2019_China:Jiangxi_2019-11-22	2019/11/22	China:Jiangxi
28	AS213/NC/CHN/2016_China:Jiangxi_2016-04-26	2016/4/26	China:Jiangxi
29	AS224/NC/CHN/2015_China:Jiangxi_2015-09-25	2015/9/25	China:Jiangxi
30	AS231/NC/CHN/2017_China:Jiangxi_2017-04-25	2017/4/25	China:Jiangxi
31	AS269/NC/CHN/2016_China:Jiangxi_2016-10-15	2016/10/15	China:Jiangxi
32	AS301/NC/CHN/2017_China:Jiangxi_2017-06-10	2017/6/10	China:Jiangxi
33	AS313/NC/CHN/2015_China:Jiangxi_2015-05-26	2015/5/26	China:Jiangxi
34	AS314/NC/CHN/2013_China:Jiangxi_2013-06-13	2013/6/13	China:Jiangxi
35	AS351/NC/CHN/2017_China:Jiangxi_2017-09-19	2017/9/19	China:Jiangxi
36	AS359/NC/CHN/2017_China:Jiangxi_2017-06-30	2017/6/30	China:Jiangxi
37	AS360/NC/CHN/2017_China:Jiangxi_2017-07-03	2017/7/3	China:Jiangxi
38	AS362/NC/CHN/2017_China:Jiangxi_2017-07-06	2017/7/6	China:Jiangxi
39	AS378/NC/CHN/2017_China:Jiangxi_2017-07-06	2017/7/6	China:Jiangxi
40	AS383/NC/CHN/2017_China:Jiangxi_2017-07-08	2017/7/8	China:Jiangxi
41	AS392/NC/CHN/2016_China:Jiangxi_2016-06-27	2016/6/27	China:Jiangxi
42	AS406/NC/CHN/2013_China:Jiangxi_2013-07-07	2013/7/7	China:Jiangxi
43	AS409/NC/CHN/2013_China:Jiangxi_2013-06-22	2013/6/22	China:Jiangxi
44	AS411/NC/CHN/2016_China:Jiangxi_2016-06-27	2016/6/27	China:Jiangxi
45	AS413/NC/CHN/2016_China:Jiangxi_2016-04-27	2016/4/27	China:Jiangxi
46	AS421/NC/CHN/2013_China:Jiangxi_2013-04-10	2013/4/10	China:Jiangxi

47	AS425/NC/CHN/2015_China:Jiangxi_2015-07-22	2015/7/22	China:Jiangxi
48	AS448/NC/CHN/2018_China:Jiangxi_2018-08-23	2018/8/23	China:Jiangxi
49	AS479/NC/CHN/2017_China:Jiangxi_2017-10-20	2017/10/20	China:Jiangxi
50	AS492/NC/CHN/2013_China:Jiangxi_2013-06-11	2013/6/11	China:Jiangxi
51	AS496/NC/CHN/2016_China:Jiangxi_2016-08-18	2016/8/18	China:Jiangxi
52	AS497/NC/CHN/2013_China:Jiangxi_2013-08-18	2013/8/18	China:Jiangxi
53	AS527/NC/CHN/2016_China:Jiangxi_2016-08-18	2016/8/18	China:Jiangxi
54	AS620/NC/CHN/2015_China:Jiangxi_2015-11-10	2015/11/10	China:Jiangxi
55	AS623/NC/CHN/2015_China:Jiangxi_2015-11-19	2015/11/19	China:Jiangxi
56	AS625/NC/CHN/2015_China:Jiangxi_2015-04-19	2015/4/19	China:Jiangxi
57	AS685/NC/CHN/2016_China:Jiangxi_2016-11-16	2016/11/16	China:Jiangxi
58	KJ641623.1_China_2013-06-28	2013/6/28	China
59	KP009574.1_China:Fuyang_2013-12-20	2013/12/20	China:Fuyang
60	KP009575.1_China:Fuyang_2013-12-20	2013/12/20	China:Fuyang
61	KP009576.1_China:Fuyang_2013-12-20	2013/12/20	China:Fuyang
62	KP009577.1_China:Fuyang_2013-12-20	2013/12/20	China:Fuyang
63	KP009578.1_China:Fuyang_2013-12-20	2013/12/20	China:Fuyang
64	KP009579.1_China:Fuyang_2013-12-20	2013/12/20	China:Fuyang
65	KP009580.1_China:Fuyang_2013-12-20	2013/12/20	China:Fuyang
66	KP009581.1_China:Fuyang_2013-12-20	2013/12/20	China:Fuyang
67	KT588920.1_China_2014-03-07	2014/3/7	China
68	KU578126.1_China_2014-06-06	2014/6/6	China
69	KU578127.1_China_2014-04-10	2014/4/10	China
70	KU578128.1_China_2014-04-02	2014/4/2	China
71	KU578129.1_China_2014-06-05	2014/6/5	China
72	KU578130.1_China_2014-04-29	2014/4/29	China
73	KU578131.1_China_2014-05-01	2014/5/1	China
74	KU578132.1_China_2014-04-17	2014/4/17	China
75	KU578133.1_China_2014-05-21	2014/5/21	China
76	KU578134.1_China_2014-04-07	2014/4/7	China
77	KU578135.1_China_2014-04-04	2014/4/4	China
78	KU578136.1_China_2014-04-03	2014/4/3	China
79	KX430803.1_Viet_Nam_2014-01-01	2014/1/1	Viet_Nam
80	KX430804.1_Viet_Nam_2014-01-01	2014/1/1	Viet_Nam
81	KX430805.1_Viet_Nam_2014-01-01	2014/1/1	Viet_Nam
82	KX430806.1_Viet_Nam_2014-01-01	2014/1/1	Viet_Nam
83	KX430807.1_Viet_Nam_2014-01-01	2014/1/1	Viet_Nam
84	KX430808.1_Viet_Nam_2014-01-01	2014/1/1	Viet_Nam
85	KX430810.1_Viet_Nam_2014-01-01	2014/1/1	Viet_Nam
86	KX595287.1_China_2013-06-01	2013/6/1	China
87	KX595288.1_China_2014-04-01	2014/4/1	China
88	KX595289.1_China_2014-04-01	2014/4/1	China
89	KX595290.1_China_2015-05-01	2015/5/1	China
90	KY271944.1_USA_2016-05-26	2016/5/26	USA
91	KY272007.1_China_2014-04-10	2014/4/10	China
92	KY272008.1_China_2014-04-03	2014/4/3	China
93	KY272009.1_China_2014-04-29	2014/4/29	China
94	KY272010.1_China_2014-06-06	2014/6/6	China

95	LC483986.1_Viet_Nam_2011-01-01	2011/1/1	Viet_Nam
96	LC483987.1_Viet_Nam_2013-01-01	2013/1/1	Viet_Nam
97	LC483988.1_Viet_Nam_2016-01-01	2016/1/1	Viet_Nam
98	LT719056.1_Madagascar_2011-01-01	2011/1/1	Madagascar
99	LT719057.1_Madagascar_2011-01-01	2011/1/1	Madagascar
100	LT719058.1_Madagascar_2011-01-01	2011/1/1	Madagascar
101	LT719059.1_Madagascar_2011-01-01	2011/1/1	Madagascar
102	LT719060.1_Madagascar_2011-01-01	2011/1/1	Madagascar
103	MF422531.1_Taiwan_2008-01-01	2008/1/1	Taiwan
104	MF422532.1_Taiwan_2008-01-01	2008/1/1	Taiwan
105	MH111058.1_Australia_2017-08-18	2017/8/18	Australia
106	MH111059.1_Australia_2017-02-22	2017/2/22	Australia
107	MH111060.1_Australia_2016-12-13	2016/12/13	Australia
108	MH111061.1_Australia_2016-11-04	2016/11/4	Australia
109	MH111062.1_Australia_2016-10-22	2016/10/22	Australia
110	MH111063.1_Australia_2017-04-02	2017/4/2	Australia
111	MH111064.1_Australia_2017-04-14	2017/4/14	Australia
112	MH111065.1_Australia_2017-03-12	2017/3/12	Australia
113	MH111066.1_Australia_2017-03-29	2017/3/29	Australia
114	MH118023.1_India_2017-01-01	2017/1/1	India
115	MH118033.1_India_2017-01-01	2017/1/1	India
116	MH118034.1_India_2017-01-01	2017/1/1	India
117	MH118036.1_India_2017-01-01	2017/1/1	India
118	MH118037.1_India_2017-01-01	2017/1/1	India
119	MH118039.1_India_2017-01-01	2017/1/1	India
120	MH118040.1_India_2016-01-01	2016/1/1	India
121	MH118042.1_India_2015-01-01	2015/1/1	India
122	MH118043.1_India_2015-01-01	2015/1/1	India
123	MH118044.1_India_2013-01-01	2013/1/1	India
124	MH118045.1_India_2013-01-01	2013/1/1	India
125	MH118046.1_India_2013-01-01	2013/1/1	India
126	MH118047.1_India_2013-01-01	2013/1/1	India
127	MH118048.1_India_2013-01-01	2013/1/1	India
128	MH118049.1_India_2013-01-01	2013/1/1	India
129	MH118050.1_India_2013-01-01	2013/1/1	India
130	MH118051.1_India_2013-01-01	2013/1/1	India
131	MH118052.1_India_2013-01-01	2013/1/1	India
132	MH118053.1_India_2012-01-01	2012/1/1	India
133	MH118055.1_India_2012-01-01	2012/1/1	India
134	MH118056.1_India_2011-01-01	2011/1/1	India
135	MH118057.1_India_2011-01-01	2011/1/1	India
136	MH118058.1_India_2011-01-01	2011/1/1	India
137	MH118059.1_India_2011-01-01	2011/1/1	India
138	MH118061.1_India_2011-01-01	2011/1/1	India
139	MH118062.1_India_2011-01-01	2011/1/1	India
140	MH118065.1_India_2011-01-01	2011/1/1	India
141	MH118066.1_India_2011-01-01	2011/1/1	India
142	MH118067.1_India_2011-01-01	2011/1/1	India

143	MH118068.1_India_2011-01-01	2011/1/1	India
144	MH118069.1_India_2011-01-01	2011/1/1	India
145	MH118070.1_India_2011-01-01	2011/1/1	India
146	MH118071.1_India_2011-01-01	2011/1/1	India
147	MH118072.1_India_2010-01-01	2010/1/1	India
148	MH118073.1_India_2010-01-01	2010/1/1	India
149	MH118074.1_India_2010-01-01	2010/1/1	India
150	MH118075.1_India_2010-01-01	2010/1/1	India
151	MH118076.1_India_2010-01-01	2010/1/1	India
152	MH118078.1_India_2010-01-01	2010/1/1	India
153	MH118079.1_India_2010-01-01	2010/1/1	India
154	MH118080.1_India_2010-01-01	2010/1/1	India
155	MH118081.1_India_2010-01-01	2010/1/1	India
156	MH118082.1_India_2010-01-01	2010/1/1	India
157	MH118083.1_India_2010-01-01	2010/1/1	India
158	MH118086.1_India_2009-01-01	2009/1/1	India
159	MH118088.1_India_2009-01-01	2009/1/1	India
160	MH118089.1_India_2009-01-01	2009/1/1	India
161	MH144590.1_India_2015-01-01	2015/1/1	India
162	MH144591.1_India_2011-01-01	2011/1/1	India
163	MH144592.1_India_2013-01-01	2013/1/1	India
164	MH144593.1_India_2017-01-01	2017/1/1	India
165	MH144594.1_India_2017-01-01	2017/1/1	India
166	MH144595.1_India_2011-01-01	2011/1/1	India
167	MH144596.1_India_2013-01-01	2013/1/1	India
168	MH144597.1_India_2017-01-01	2017/1/1	India
169	MH144599.1_India_2013-01-01	2013/1/1	India
170	MH144603.1_India_2013-01-01	2013/1/1	India
171	MK301475.1_China_2017-10-01	2017/10/1	China
172	MK301476.1_China_2018-09-01	2018/9/1	China
173	MK301477.1_China_2018-09-01	2018/9/1	China
174	MK301478.1_China_2018-10-01	2018/10/1	China
175	MK301479.1_China_2018-10-01	2018/10/1	China
176	MK645898.1_China_2012-04-01	2012/4/1	China
177	MK791147.1_China_2018-08-27	2018/8/27	China
178	MK814854.1_China_2018-06-16	2018/6/16	China
179	MK814855.1_China_2018-06-26	2018/6/26	China
180	MK867822.1_China_2017-10-01	2017/10/1	China
181	MK867823.1_China_2017-10-01	2017/10/1	China
182	MK867824.1_China_2017-10-01	2017/10/1	China
183	MK867825.1_China_2009-05-01	2009/5/1	China
184	MK867826.1_China_2010-09-01	2010/9/1	China
185	MK867827.1_China_2015-11-01	2015/11/1	China
186	MK867828.1_China_2013-10-01	2013/10/1	China
187	MK867829.1_China_2015-11-01	2015/11/1	China
188	MK965226.1_China_2018-07-16	2018/7/16	China
189	MK965229.1_China_2018-07-16	2018/7/16	China
190	MK965239.1_China_2018-05-27	2018/5/27	China

191	MK965241.1_China_2018-07-04	2018/7/4	China
192	MK967667.1_China_2015-05-20	2015/5/20	China
193	MK967668.1_China_2014-06-05	2014/6/5	China
194	MK967669.1_China_2014-04-04	2014/4/4	China
195	MK967670.1_China_2014-04-17	2014/4/17	China
196	MK967671.1_China_2014-05-01	2014/5/1	China
197	MT263729.2_China_2009-10-01	2009/10/1	China
198	MT347977.1_USA_2014-01-01	2014/1/1	USA
199	MT828546.1_China_2018-09-01	2018/9/1	China
200	MT828547.1_China_2018-11-01	2018/11/1	China
201	MT828548.1_China_2018-12-01	2018/12/1	China
202	MT828549.1_China_2018-12-01	2018/12/1	China
203	MW929238.1_China_2017-10-01	2017/10/1	China
204	MW929239.1_China_2018-07-01	2018/7/1	China
205	MW929240.1_China_2019-05-01	2019/5/1	China
206	MW929241.1_China_2017-12-01	2017/12/1	China
207	MW929242.1_China_2019-03-01	2019/3/1	China
208	MW929243.1_China_2019-03-01	2019/3/1	China
209	MW929244.1_China_2019-05-01	2019/5/1	China
210	MW929245.1_China_2019-05-01	2019/5/1	China
211	MW929246.1_China_2019-06-01	2019/6/1	China
212	MW929247.1_China_2019-06-01	2019/6/1	China
213	MW929248.1_China_2019-07-01	2019/7/1	China
214	MW929249.1_China_2019-09-01	2019/9/1	China
215	MW929250.1_China_2019-09-01	2019/9/1	China
216	MW929251.1_China_2018-04-01	2018/4/1	China
217	MW929252.1_China_2019-08-01	2019/8/1	China
218	MW929253.1_China_2019-08-01	2019/8/1	China
219	MW929254.1_China_2019-08-01	2019/8/1	China
220	MW929255.1_China_2019-08-01	2019/8/1	China
221	MW929256.1_China_2018-08-01	2018/8/1	China
222	MW929257.1_China_2018-08-01	2018/8/1	China
223	MW929258.1_China_2019-06-01	2019/6/1	China
224	MW929259.1_China_2019-10-01	2019/10/1	China
225	MW929260.1_China_2017-11-01	2017/11/1	China
226	MW929261.1_China_2019-06-01	2019/6/1	China
227	MW929262.1_China_2019-07-01	2019/7/1	China
228	MW929263.1_China_2019-07-01	2019/7/1	China
229	MW929264.1_China_2019-07-01	2019/7/1	China
230	MW929265.1_China_2019-07-01	2019/7/1	China
231	MW929266.1_China_2018-06-01	2018/6/1	China
232	MW929267.1_China_2018-06-01	2018/6/1	China
233	MW929268.1_China_2018-04-01	2018/4/1	China
234	MW929269.1_China_2018-06-01	2018/6/1	China
235	MW929270.1_China_2019-04-01	2019/4/1	China
236	MW929271.1_China_2019-07-01	2019/7/1	China
237	MW929272.1_China_2019-08-01	2019/8/1	China
238	MW929273.1_China_2019-10-01	2019/10/1	China

239	MW929274.1_China_2017-02-01	2017/2/1	China
240	MW929275.1_China_2018-05-01	2018/5/1	China
241	MW929276.1_China_2019-05-01	2019/5/1	China
242	MW929277.1_China_2019-06-01	2019/6/1	China
243	MW929278.1_China_2018-04-01	2018/4/1	China
244	MW929279.1_China_2018-08-01	2018/8/1	China
245	MW929280.1_China_2018-08-01	2018/8/1	China
246	MW929281.1_China_2016-08-01	2016/8/1	China
247	MW929282.1_China_2016-09-01	2016/9/1	China
248	MW929283.1_China_2017-04-01	2017/4/1	China
249	MW929284.1_China_2017-05-01	2017/5/1	China
250	MW929285.1_China_2018-04-01	2018/4/1	China
251	MW929286.1_China_2018-05-01	2018/5/1	China
252	MW929287.1_China_2018-07-01	2018/7/1	China
253	MW929288.1_China_2019-06-01	2019/6/1	China
254	MW929289.1_China_2017-10-01	2017/10/1	China
255	MW929290.1_China_2018-07-01	2018/7/1	China
256	MW929291.1_China_2019-04-01	2019/4/1	China
257	MW929292.1_China_2019-06-01	2019/6/1	China
258	MW929293.1_China_2019-07-01	2019/7/1	China
259	MW929294.1_China_2019-09-01	2019/9/1	China
260	MW929295.1_China_2020-01-01	2020/1/1	China
261	MW929296.1_China_2020-01-01	2020/1/1	China
262	MW929297.1_China_2020-01-01	2020/1/1	China
263	MW929298.1_China_2019-06-01	2019/6/1	China
264	MW929299.1_China_2019-07-01	2019/7/1	China
265	MW929300.1_China_2019-08-01	2019/8/1	China
266	MW929301.1_China_2019-09-01	2019/9/1	China
267	MZ491033.1_China_2019-03-01	2019/3/1	China
268	MZ491034.1_China_2019-03-01	2019/3/1	China
269	OK570247.1_Madagascar_2011-01-01	2011/1/1	Madagascar
270	OK570248.1_Madagascar_2011-01-01	2011/1/1	Madagascar
271	OK570250.1_Madagascar_2011-01-01	2011/1/1	Madagascar
272	OK570252.1_Madagascar_2011-01-01	2011/1/1	Madagascar
273	OK570260.1_Madagascar_2011-01-01	2011/1/1	Madagascar
274	OR095105.1_China:Zhejiang_2017-05-25	2017/5/25	China:Zhejiang
275	OR095106.1_China:Zhejiang_2017-05-19	2017/5/19	China:Zhejiang
276	OR095107.1_China:Zhejiang_2017-07-03	2017/7/3	China:Zhejiang
277	OR095108.1_China:Zhejiang_2020-02-28	2020/2/28	China:Zhejiang
278	OR095109.1_China:Zhejiang_2019-06-06	2019/6/6	China:Zhejiang
279	OR095110.1_China:Zhejiang_2018-04-06	2018/4/6	China:Zhejiang
280	OR095111.1_China:Zhejiang_2018-05-20	2018/5/20	China:Zhejiang
281	OR095112.1_China:Zhejiang_2018-05-15	2018/5/15	China:Zhejiang
282	OR095113.1_China:Zhejiang_2017-07-04	2017/7/4	China:Zhejiang
283	OR095114.1_China:Zhejiang_2017-06-07	2017/6/7	China:Zhejiang
284	OR095115.1_China:Zhejiang_2018-01-15	2018/1/15	China:Zhejiang
285	OR095116.1_China:Zhejiang_2018-01-11	2018/1/11	China:Zhejiang
286	OR095117.1_China:Zhejiang_2018-02-10	2018/2/10	China:Zhejiang

287	OR095118.1_China:Zhejiang_2018-05-10	2018/5/10	China:Zhejiang
288	OR095119.1_China:Zhejiang_2017-05-03	2017/5/3	China:Zhejiang
289	OR095120.1_China:Zhejiang_2017-07-13	2017/7/13	China:Zhejiang
290	OR095121.1_China:Zhejiang_2017-07-13	2017/7/13	China:Zhejiang
291	OR095122.1_China:Zhejiang_2017-07-13	2017/7/13	China:Zhejiang
292	OR095123.1_China:Zhejiang_2017-04-07	2017/4/7	China:Zhejiang
293	OR095124.1_China:Zhejiang_2018-03-18	2018/3/18	China:Zhejiang
294	OR095125.1_China:Zhejiang_2021-01-09	2021/1/9	China:Zhejiang
295	OR095126.1_China:Zhejiang_2017-05-26	2017/5/26	China:Zhejiang
296	OR095127.1_China:Zhejiang_2017-05-25	2017/5/25	China:Zhejiang
297	OR095128.1_China:Zhejiang_2021-05-26	2021/5/26	China:Zhejiang
298	OR095129.1_China:Zhejiang_2022-02-13	2022/2/13	China:Zhejiang
299	OR095130.1_China:Zhejiang_2020-08-04	2020/8/4	China:Zhejiang
300	OR095131.1_China:Zhejiang_2021-05-20	2021/5/20	China:Zhejiang
301	OR095132.1_China:Zhejiang_2021-05-06	2021/5/6	China:Zhejiang
302	OR095133.1_China:Zhejiang_2021-05-08	2021/5/8	China:Zhejiang
303	OR095134.1_China:Zhejiang_2021-05-11	2021/5/11	China:Zhejiang
304	OR095135.1_China:Zhejiang_2021-05-26	2021/5/26	China:Zhejiang
305	OR095136.1_China:Zhejiang_2021-05-24	2021/5/24	China:Zhejiang
306	OR095137.1_China:Zhejiang_2021-05-18	2021/5/18	China:Zhejiang
307	OR095138.1_China:Zhejiang_2021-06-16	2021/6/16	China:Zhejiang
308	OR095139.1_China:Zhejiang_2021-04-26	2021/4/26	China:Zhejiang
309	OR095140.1_China:Zhejiang_2021-06-22	2021/6/22	China:Zhejiang
310	OR095141.1_China:Zhejiang_2019-06-21	2019/6/21	China:Zhejiang
311	OR095142.1_China:Zhejiang_2019-03-27	2019/3/27	China:Zhejiang
312	OR095143.1_China:Zhejiang_2019-02-06	2019/2/6	China:Zhejiang
313	OR095144.1_China:Zhejiang_2019-01-08	2019/1/8	China:Zhejiang
314	OR095145.1_China:Zhejiang_2019-03-16	2019/3/16	China:Zhejiang
315	OR095146.1_China:Zhejiang_2021-06-12	2021/6/12	China:Zhejiang
316	OR095147.1_China:Zhejiang_2020-09-17	2020/9/17	China:Zhejiang
317	OR095148.1_China:Zhejiang_2020-09-24	2020/9/24	China:Zhejiang
318	OR095149.1_China:Zhejiang_2020-08-24	2020/8/24	China:Zhejiang
319	OR095150.1_China:Zhejiang_2020-08-14	2020/8/14	China:Zhejiang
320	OR095151.1_China:Zhejiang_2021-06-25	2021/6/25	China:Zhejiang
321	OR095152.1_China:Zhejiang_2021-07-01	2021/7/1	China:Zhejiang
322	OR095153.1_China:Zhejiang_2022-07-29	2022/7/29	China:Zhejiang
323	OR095154.1_China:Zhejiang_2022-07-06	2022/7/6	China:Zhejiang
324	OR095155.1_China:Zhejiang_2018-10-01	2018/10/1	China:Zhejiang
325	OR095156.1_China:Zhejiang_2018-07-06	2018/7/6	China:Zhejiang
326	OR095157.1_China:Zhejiang_2019-06-01	2019/6/1	China:Zhejiang
327	OR828446.1_China:Changsha_2019-04-16	2019/4/16	China:Changsha
328	PP078926.1_China:Guangdong_2016-06-29	2016/6/29	China:Guangdong
329	PP078927.1_China:Guangdong_2016-06-28	2016/6/28	China:Guangdong
330	PP078928.1_China:Guangdong_2019-03-16	2019/3/16	China:Guangdong
331	PP078929.1_China:Guangdong_2018-05-20	2018/5/20	China:Guangdong
332	PP078930.1_China:Guangdong_2018-06-06	2018/6/6	China:Guangdong
333	PP078931.1_China:Guangdong_2018-05-30	2018/5/30	China:Guangdong
334	PP078932.1_China:Guangdong_2018-07-27	2018/7/27	China:Guangdong

335	PP078933.1_China:Guangdong_2018-07-09	2018/7/9	China:Guangdong
336	PP078934.1_China:Guangdong_2018-05-29	2018/5/29	China:Guangdong
337	PP078935.1_China:Guangdong_2018-06-05	2018/6/5	China:Guangdong
338	PP078936.1_China:Guangdong_2017-07-12	2017/7/12	China:Guangdong
339	PP078937.1_China:Guangdong_2015-06-11	2015/6/11	China:Guangdong
340	PP078938.1_China:Guangdong_2015-08-08	2015/8/8	China:Guangdong
341	PP078939.1_China:Guangdong_2016-05-24	2016/5/24	China:Guangdong
342	PP078940.1_China:Guangdong_2016-05-03	2016/5/3	China:Guangdong
343	PP078941.1_China:Guangdong_2016-05-10	2016/5/10	China:Guangdong
344	PP078942.1_China:Guangdong_2017-06-15	2017/6/15	China:Guangdong
345	PP078943.1_China:Guangdong_2016-05-16	2016/5/16	China:Guangdong
346	PP078944.1_China:Guangdong_2018-05-25	2018/5/25	China:Guangdong
347	PP078945.1_China:Guangdong_2018-06-01	2018/6/1	China:Guangdong
348	PP078946.1_China:Guangdong_2018-06-13	2018/6/13	China:Guangdong
349	PP078947.1_China:Guangdong_2021-01-15	2021/1/15	China:Guangdong
350	PP078948.1_China:Guangdong_2021-02-20	2021/2/20	China:Guangdong
351	PP078949.1_China:Guangdong_2017-06-22	2017/6/22	China:Guangdong
352	PP078950.1_China:Guangdong_2015-11-02	2015/11/2	China:Guangdong
353	PP078951.1_China:Guangdong_2017-06-09	2017/6/9	China:Guangdong
354	PP078952.1_China:Guangdong_2015-04-15	2015/4/15	China:Guangdong
355	PP078953.1_China:Guangdong_2015-06-16	2015/6/16	China:Guangdong
356	PP078954.1_China:Guangdong_2010-06-15	2010/6/15	China:Guangdong
357	PP078955.1_China:Guangdong_2015-07-13	2015/7/13	China:Guangdong
358	PP078956.1_China:Guangdong_2015-09-13	2015/9/13	China:Guangdong
359	PP078957.1_China:Guangdong_2015-05-21	2015/5/21	China:Guangdong
360	PP078958.1_China:Guangdong_2018-06-05	2018/6/5	China:Guangdong
361	PP078959.1_China:Guangdong_2016-06-14	2016/6/14	China:Guangdong
362	PP078960.1_China:Guangdong_2016-06-14	2016/6/14	China:Guangdong
363	PP078961.1_China:Guangdong_2017-06-02	2017/6/2	China:Guangdong
364	PP078962.1_China:Guangdong_2018-05-14	2018/5/14	China:Guangdong
365	PP078963.1_China:Guangdong_2018-06-01	2018/6/1	China:Guangdong
366	PP078964.1_China:Guangdong_2020-09-19	2020/9/19	China:Guangdong
367	PP078965.1_China:Guangdong_2020-10-05	2020/10/5	China:Guangdong
368	PP078966.1_China:Guangdong_2021-01-13	2021/1/13	China:Guangdong
369	PP078967.1_China:Guangdong_2021-01-13	2021/1/13	China:Guangdong
370	PP078968.1_China:Guangdong_2020-11-24	2020/11/24	China:Guangdong
371	PP078969.1_China:Guangdong_2021-01-05	2021/1/5	China:Guangdong
372	PP078970.1_China:Guangdong_2021-01-12	2021/1/12	China:Guangdong
373	PP078971.1_China:Guangdong_2019-08-14	2019/8/14	China:Guangdong
374	PP078972.1_China:Guangdong_2019-10-04	2019/10/4	China:Guangdong
375	PP078973.1_China:Guangdong_2018-06-02	2018/6/2	China:Guangdong
376	PP078974.1_China:Guangdong_2018-06-04	2018/6/4	China:Guangdong
377	PP078975.1_China:Guangdong_2018-06-04	2018/6/4	China:Guangdong
378	PP078976.1_China:Guangdong_2019-04-25	2019/4/25	China:Guangdong
379	PP078977.1_China:Guangdong_2017-07-06	2017/7/6	China:Guangdong
380	PP078978.1_China:Guangdong_2017-08-15	2017/8/15	China:Guangdong
381	PP078979.1_China:Guangdong_2018-05-29	2018/5/29	China:Guangdong
382	PP078980.1_China:Guangdong_2018-06-14	2018/6/14	China:Guangdong

383	PP078981.1_China:Guangdong_2018-05-09	2018/5/9	China:Guangdong
384	PP078982.1_China:Guangdong_2018-05-23	2018/5/23	China:Guangdong
385	PP078983.1_China:Guangdong_2018-06-05	2018/6/5	China:Guangdong
386	PP078984.1_China:Guangdong_2018-05-23	2018/5/23	China:Guangdong
387	PP078985.1_China:Guangdong_2018-05-08	2018/5/8	China:Guangdong
388	PP078986.1_China:Guangdong_2018-05-25	2018/5/25	China:Guangdong
389	PP078987.1_China:Guangdong_2018-05-07	2018/5/7	China:Guangdong
390	PP078988.1_China:Guangdong_2018-06-05	2018/6/5	China:Guangdong
391	PP078989.1_China:Guangdong_2018-06-30	2018/6/30	China:Guangdong
392	PP078990.1_China:Guangdong_2018-06-05	2018/6/5	China:Guangdong
393	PP078991.1_China:Guangdong_2018-06-18	2018/6/18	China:Guangdong
394	PP078992.1_China:Guangdong_2018-06-04	2018/6/4	China:Guangdong
395	PP078993.1_China:Guangdong_2019-10-09	2019/10/9	China:Guangdong
396	PP078994.1_China:Guangdong_2019-10-09	2019/10/9	China:Guangdong
397	PP078995.1_China:Guangdong_2021-01-07	2021/1/7	China:Guangdong
398	PP078996.1_China:Guangdong_2020-10-17	2020/10/17	China:Guangdong
399	PP078997.1_China:Guangdong_2020-12-08	2020/12/8	China:Guangdong
400	PP078998.1_China:Guangdong_2019-04-18	2019/4/18	China:Guangdong
401	PP078999.1_China:Guangdong_2020-12-17	2020/12/17	China:Guangdong
402	PP079000.1_China:Guangdong_2020-11-06	2020/11/6	China:Guangdong
403	PP079001.1_China:Guangdong_2021-01-05	2021/1/5	China:Guangdong
404	PP208912.1_China:Guangdong_2016-05-02	2016/5/2	China:Guangdong
405	PP208913.1_China:Guangdong_2018-05-28	2018/5/28	China:Guangdong

Supplementary Table S3 Summary of CVA10 recombination events detected by RDP

Isolate	Recombination event No.	Breakpoint	Major parent	Minor parent	Detection methods and *P*-values						
					RDP	Geneconv	Bootscan	Maxchi	Chimaera	SiScan	3Seq
AS479/2017	1	6510-7164	2015-XMCDC-241-CVA10	A10 strain 61217-2276	3.19E-02	6.22E-05	1.36E-04	NS	NS	5.16E-04	2.16E-03
AS177/2018	1	6515-7164	2015-XMCDC-241-CVA10	A10 strain 61217-2276	4.72E-03	NS	2.98E-05	7.64E-04	3.27E-03	5.62E-05	NS
AS207/2019	1	6532-7164	2015-XMCDC-241-CVA10	A10 strain 61217-2276	2.16E-03	3.45E-03	1.94E-03	3.46E-05	NS	3.26E-04	NS

NS represented no significant *P*-value was recorded for recombination event using this method.

Isolate 2015-XMCDC-241-CVA10 (Genbank accession no: KX768167) and CVA10-G-A10 strain 61217-2276 (Genbank accession no: MF422532) were chosen to be the reference sequences for recombination analysis. CVA10, Coxsackievirus A10.
